# Exposure to Secreted Bacterial Factors Promotes HIV-1 Replication in CD4^+^ T Cells

**DOI:** 10.1128/spectrum.04313-22

**Published:** 2023-02-28

**Authors:** M. Znaidia, Y. de Souza-Angelo, S. Létoffé, I. Staropoli, L. Grzelak, J. M. Ghigo, O. Schwartz, N. Casartelli

**Affiliations:** a Institut Pasteur, Université Paris-Cité, UMR CNRS 3569, Virus and Immunity Unit, Paris, France; b Vaccine Research Institute, Créteil, France; c Institut Pasteur, Université Paris-Cité, UMR CNRS 6047, Genetics of Biofilms Laboratory, Paris, France; Kumamoto University

**Keywords:** HIV-1, T cells, Gram-negative bacteria, Gram-positive bacteria

## Abstract

Microbial translocation is associated with systemic immune activation in HIV-1 disease. Circulating T cells can encounter microbial products in the bloodstream and lymph nodes, where viral replication takes place. The mechanisms by which bacteria contribute to HIV-associated pathogenesis are not completely deciphered. Here, we examined how bacteria may impact T cell function and viral replication. We established cocultures between a panel of live bacteria and uninfected or HIV-1-infected activated peripheral blood CD4-positive (CD4^+^) T cells. We show that some bacteria, such as Escherichia coli and Acinetobacter baumannii, sustain lymphocyte activation and enhance HIV-1 replication. Bacteria secrete soluble factors that upregulate CD25 and ICAM-1 cell surface levels and activate NF-κB nuclear translocation. Our data also demonstrate that CD25 polarizes at the virological synapse, suggesting a previously unappreciated role of CD25 during viral replication. These findings highlight how interactions between bacterial factors and T cells may promote T cell activation and HIV-1 replication.

**IMPORTANCE** People living with HIV suffer from chronic immune activation despite effective antiretroviral therapy. Early after infection, HIV-1 actively replicates in the gut, causing the breakage of the intestinal epithelial barrier and microbial translocation. Microbial translocation and chronic immune activation have been proven linked; however, gaps in our knowledge on how bacteria contribute to the development of HIV-related diseases remain. Whether T cells in the peripheral blood react to bacterial products and how this affects viral replication are unknown. We show that some bacteria enriched in people living with HIV activate T cells and favor HIV-1’s spread. Bacteria release soluble factors that cause the overexpression of cellular molecules related to their activation state. T cells overexpressing these molecules also replicate HIV-1 more efficiently. These results help us learn more about how HIV-1, T cells, and bacteria interact with each other, as well as the mechanisms behind chronic immune activation.

## INTRODUCTION

HIV-1 infection is characterized by a progressive loss of peripheral blood CD4-positive (CD4^+^) T cells, an imbalance in CD4^+^ T-cell homeostasis, as well as a gradual deterioration of immune function. HIV-1 infects cells either as a cell-free virus or via cell-to-cell viral transmission (1). The latter is more efficient and achieved by the formation of contacts called virological synapses (VS) between a donor infected cell and one or multiple target cells (2–4). Cell-to-cell HIV-1 transmission is thought to happen mostly in lymph nodes, where the high cell density favors contacts between cells and where clusters of infected cells have been visualized (2). HIV-1 enters cells through the CD4 receptor and one coreceptor, CCR5 or CXCR4. Initial transmission events are mostly caused by CCR5-using viruses circulating in both early and chronic infections (5, 6). CXCR4-dependent viruses appear later when the immune system is weakened (7–9). Together with CD4 and coreceptors, cellular adhesion molecules such as ICAM-1 and ICAM-3 contribute to the formation and stabilization of VS (10). ICAM-1 and its ligand, LFA-1, also promote cell-free virus infectivity (11–13).

The activation status of T cells is crucial for productive HIV replication. Resting peripheral blood T cells are poorly permissive to HIV infection; the virus can enter and integrate but does not progress further in its replication cycle unless a mitogenic stimulus is present (14–16). In contrast, gut lamina propria CD4^+^ T cells have a higher basal activation state that renders them susceptible to infection without exogenous stimuli (17).

Early after HIV-1 infection, lamina propria CD4^+^ T cells are drastically reduced, triggering a cascade of events leading to gut dysbiosis, reducing the abundance of beneficial gut symbionts, and causing the disruption of the intestinal epithelium’s tight junctions. This leads to an increased sensitivity of people living with HIV-1 to opportunistic bacterial pathogens, including Staphylococcus aureus, Escherichia coli, Klebsiella pneumoniae, Pseudomonas aeruginosa, S. pneumoniae, Salmonella spp., Haemophilus influenzae, Mycobacterium tuberculosis, and Mycobacterium avium complex (18–26). The rupture of the intestinal epithelial barrier allows the release of commensal and pathogenic bacteria and bacterial factors into the bloodstream that quickly reach distal and peripheral lymph nodes (27, 28).

Microbial translocation and dysbiosis contribute to persistent immune activation and inflammation that may not be restored by early antiretroviral therapy (ART) (29–35). Chronic immune activation is a strong indicator of disease progression in people living with HIV (31, 32, 36, 37).

Besides the gut, other lymphoid organs are replication sites during HIV-1’s acute and chronic phases. HIV-infected T cells accumulate in lymph nodes, the central nervous system, and genitourinary tract (38, 39). Lymph nodes contain more infected cells than peripheral blood and are sites of high levels of viral replication and T-cell activation (40, 41).

The interactions between microbial products, such as pathogen-associated molecular patterns (or PAMPs) and pattern recognition receptors expressed by innate immune cells, allow microbial detection, activate innate immune responses, and induce the production of proinflammatory cytokines (42). These responses significantly contribute to the aberrant immune activation observed in chronic HIV infection (31, 43). Innate immune sensing has also been demonstrated in lymphocytes (44), and human T cells express most Toll-like receptors (TLRs) (44–47), acting as costimulatory signals and promoting T cell activation (46, 48, 49).

Circulating T cells are continuously exposed to bacterial factors in the bloodstream and when they transit in the lymph nodes, where viral transmission occurs. How T cells respond to bacterial stimuli and whether bacteria affect viral replication in peripheral blood T cells remain largely uncharacterized. By establishing cocultures between uninfected or infected T cells and bacteria, and by following them over time, we show that various bacteria sustain the activation of the primary CD4^+^ T cell and promote HIV-1 replication. We show that lymphocyte activation is triggered by bacterial factors released in the extracellular medium that induce NF-κB nuclear translocation and CD25 and ICAM-1 upregulation. Finally, we show, for the first time, the presence of CD25 at the VS, together with HIV-1 Gag proteins.

These results contribute to the understanding of the interactions between bacteria, T cells, and HIV-1. They pave the way for the identification of new mechanisms that promote HIV-1 replication and induce aberrant activation of T cells.

## RESULTS

### Escherichia coli stimulates HIV-1 replication in activated CD4^+^ T cells.

**FIG 1:**
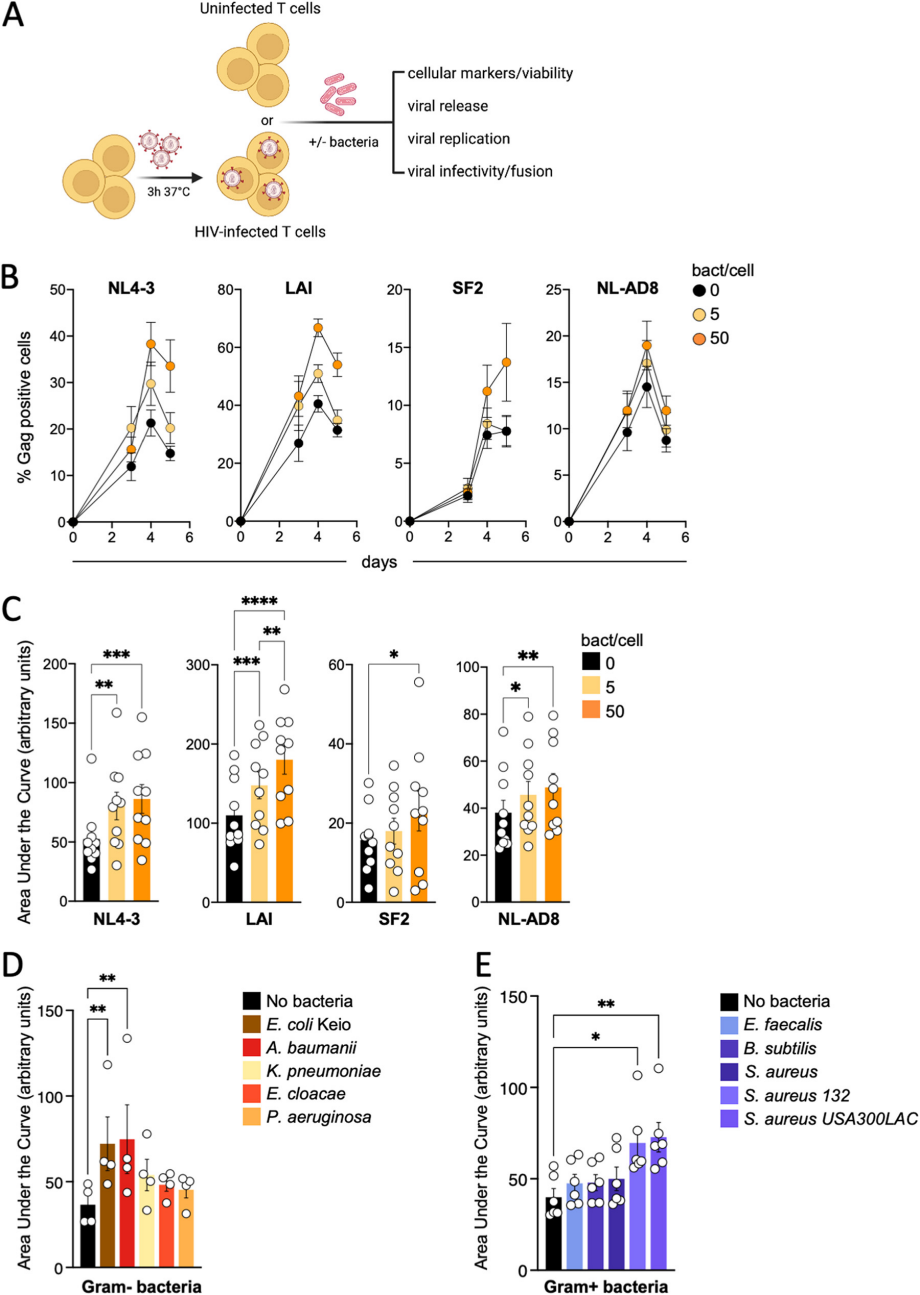
Effects of the coculture of primary CD4^+^ T cells with E. coli during HIV-1 replication. (A) Schematic overview of the experimental procedure used to study the interplay between bacteria, CD4^+^ T cells, and HIV-1. (B) Replication of the virus in untreated primary CD4^+^ T cells or cells cocultured with E. coli. Primary CD4^+^ T cells were infected with the indicated viruses and then left untreated or set in the culture in the presence of 5 or 50 bacteria/cell. Viral replication was analyzed over a week by following the appearance of Gag^+^ cells in culture by flow cytometry. Graphs represent the means ± SEMs of the viral replication in three independent donors tested in a representative experiment. (C) Analysis of the area under the curve of the viral replication as observed in untreated or bacteria-exposed T cells. The area under the curve has been calculated for each infection for each donor. Each dot corresponds to an independent donor. Cells of each donor have been left untreated or exposed to E. coli at the indicated ratios. (D and E) Primary CD4^+^ T cells were infected with NL4-3. After infection, cells were left untreated or cocultivated with the indicated bacteria at a ratio of 50 bacteria/cell. Graphs represent the area under the curve of the viral replication over a week. Each dot corresponds to an independent donor. Cells of each donor have been left untreated or exposed to the bacteria (*n* = 4 for Gram-negative bacteria; *n* = 6 for Gram-positive bacteria). Statistical differences between the different conditions have been calculated using the analysis of variance (ANOVA) paired statistical test. *, *P* ≤ 0.05; **, *P ≤ *0.01; ***, *P* ≤ 0.001; ****, *P* ≤ 0.0001.

To analyze the interplay between bacteria and activated peripheral blood CD4^+^ T cells (PBT), we developed the protocol outlined in Fig. 1 . We used the E. coli commensal strain MG1655 (50) as a representative enteric bacterium (51–54).

We first tested if E. coli bacteria could affect phytohemagglutinin-activated PBT’s growth and viability. Activated PBT were plated alone or with bacteria (5:1 and 50:1 bacteria-T cell ratios, respectively) in the presence of freshly added penicillin and streptomycin to stop bacterial growth. We then checked the T cells’ forward scatter-side scatter (FSC-SSC) flow cytometry profile and counted the number of live cells over a week. While untreated cells nearly doubled and cell growth was not affected at the 5:1 ratio, we observed, at the 50:1 ratio, a slight growth defect at day 4, but an untreated level was reached by day 6. We therefore found no significant differences in growth of untreated and bacteria-exposed T cells (see Fig. S1 in the supplemental material).

PBT were infected with the CXCR4-using strain NL4-3 (Fig. 1B and C). Using flow cytometry, we monitored the appearance of Gag^+^ cells as a readout for viral replication. NL4-3 replication peaked at 20% Gag^+^ cells on day 4 postinfection in the absence of bacteria. With bacteria, cells replicated NL4-3 more efficiently, with twice as many Gag^+^ cells at day 4 with the high bacterial dose. We assessed the impact of bacteria on the following other viral strains with different tropisms: Lai (CXCR4), SF2 (CCR5/CXCR4), and NL-AD8 (CCR5). Lai and SF2 replication reached 30% and 8% of Gag^+^ T cells, respectively, on day 4 (Fig. 1B and C). Similar to NL4-3, Lai viral replication was accelerated by both bacterial ratios. SF2 replicated more efficiently only with 50 bacteria/cell. Both bacterial ratios promoted NL-AD8 replication.

To ensure that the observed effects were not due to phytohemagglutinin-nonspecific mitogenic activity on T cells, we also used CD3/CD28/CD2 antibody-coated beads to stimulate PBT. After infection with NL4-3 or NL-AD8, PBT were left untreated or cocultured with the bacteria at a ratio of 50:1. In the presence of bacteria, NL4-3 and NL-AD8 replicated substantially more efficiently (Fig. S1).

These results indicate that coculturing infected cells with bacteria boosts replication of different viral strains independently of the mitogenic stimulus used to activate T cells.

### Activation of HIV-1 replication in primary CD4^+^ T cells is triggered by various bacteria.

To test the range of bacteria boosting viral replication, PBT were infected with NL4-3, and we selected a panel of commensal or pathogenic Gram-negative or Gram-positive bacteria (Table S1 and Fig. 1D and E) that are modulated during HIV-1 infection or may cause opportunistic infection in people living with HIV-1 (Table S1). We also included an E. coli strain close to MG1655 in which the Keio collection of nonessential gene mutations is available (BW25113, here referred to as the E. coli Keio strain [55]) as well as Enterobacter cloacae (Gram negative) and Bacillus subtilis (Gram positive), which have not been associated with HIV-1 infection. The E. coli Keio strain enhanced HIV-1 replication similarly to MG1655. Along the Gram-negative bacteria, only A. baumannii significantly increased viral spread (a 2-fold increase). However, PBT cocultured with K. pneumoniae, E. cloacae, and P. aeruginosa consistently replicated the virus slightly more efficiently, with a 1.2- to 1.5-fold increase (Fig. 1D). Among the Gram positive, we included two methicillin-resistant S. aureus (MRSA) strains, S. aureus strains 132 and USA300LAC (56, 57). S. aureus USA300 is the most common lineage found with S. aureus infections in people living with HIV-1 (58, 59). Enterococcus faecalis and B. subtilis did not enhance viral replication. While the wild-type S. aureus did not impact viral replication, both MRSA significantly enhanced viral spread (Fig. 1E).

The bacterial ability to promote viral replication in PBT is thus not ubiquitous but shared by various Gram-negative and Gram-positive bacteria.

### T cells exposed to bacteria are more permissive to viral fusion and produce more infectious viral particles.

**FIG 2:**
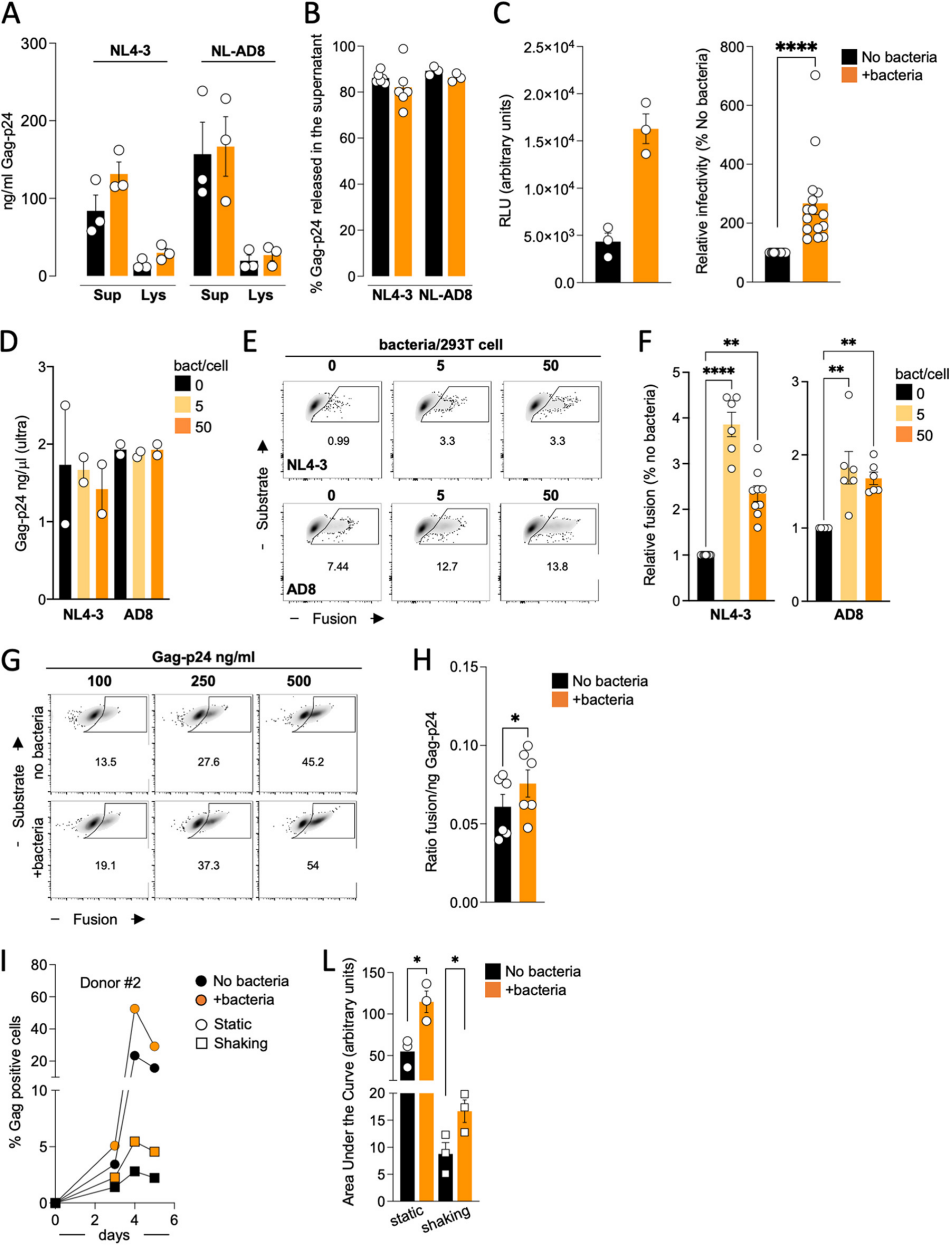
Viral particles produced in the presence of bacterial cells are more infectious, and target cells exposed to bacteria are more sensitive to viral fusion. (A) Dosage ELISA of the HIV-1 Gag-p24 in the supernatants (Sup) and cell lysates (Lys) of the primary CD4^+^ T cells infected with NL4-3 or NL-AD8 viruses and cocultivated with E. coli MG1655 or left untreated (nanograms per milliliter). One out of two independent experiments is depicted. Each dot corresponds to an independent donor that has been exposed to E. coli MG1655 or left untreated. (B) Percentage of viral release calculated as the percentage of Gag-p24 measured in the supernatant divided by the total (supernatant + cell lysate) Gag-p24. (C) Viral infectivity assay. The TZM-bl reported cells have been infected with a normalized amount of Gag-p24 produced by primary CD4^+^ T cells exposed or not to E. coli MG1655 during viral replication. (C, Left) Raw data (mean ± SEM) of the relative luciferase units (RLU) for a representative donor tested in triplicate are shown. Relative infectivity has been calculated by setting at 100% the infectivity associated with the viral particles derived from untreated cells for each donor. (C, Right) The mean ± SEM of the triplicate from 5 independent donors is presented. One sample *t* test and Wilcoxon test have been used to analyze statistical differences, ****, *P ≤ *0.0001. (D) Dosage ELISA of the amount of Gag-p24 produced by the 293T after transfection with or without E. coli. Mean ± SEM of two independent viral productions either for NL4-3 or for NL-AD8 is presented. (E) Viral particles produced in panel D were used for a viral fusion assay. Activated primary CD4^+^ T cells were used as target cells and exposed to 100 ng/mL of viral particles produced by 293T cells left untreated or exposed to 5 or 50 E. coli MG1655 per cell. One representative experiment is shown. Numbers in the density plots indicate the percentage of fusion-positive cells. (F) Relative fusion is expressed as the percentage of the fusion observed with the viruses produced by untreated 293T cells. Mean ± SEM of two independent experiments is shown. ANOVA paired test has been used to calculate statistical differences; **, *P ≤* 0.01; ****, *P ≤* 0.0001. (G) Fusion assay. Target primary CD4^+^ T cells were left untreated or exposed to E. coli MG1655 (50 bacteria per cell) 24 h before the fusion assay. Cells were then incubated with the indicated amount (nanograms per milliliter) of NL4-3-BLam-Vpr viruses for 2 h, and then the efficiency of viral fusion was evaluated by flow cytometry. One representative experiment out of two is shown. Numbers in the density plots indicate the percentage of fusion-positive cells. (H) The ratio between the percentage of fusion-positive cells and the amount of Gag-p24 has been calculated, and the mean ± SEM of two independent experiments is depicted (right). Wilcoxon matched-pair test has been used to calculate statistical differences. *, *P* ≤ 0.05. (I) Primary CD4^+^ T cells were infected and then left in culture in static position or maintained in gentle shaking with or without E. coli MG1655 in the culture. Viral replication was monitored over 1 week. One representative donor is shown. (L) The area under the curve of the viral replication has been calculated for each condition. The mean ± SEM for three independent donors is represented. Paired *t* test has been used to calculate statistical differences. *, *P ≤* 0.05.

To detail the effects of bacteria on cells and viruses, we used E. coli MG1655 as a reference bacterium. We first investigated, by enzyme-linked immunosorbent assay (ELISA), whether bacteria increased viral release from infected cells (Fig. 2A and B). We measured the proportion of Gag p24 released in the supernatant relative to the total amount of Gag p24 generated. We did not find any significant changes between T cells cocultured with bacteria and control cells. Therefore, bacteria do not stimulate viral release (Fig. 2B).

We then evaluated the infectivity of viral supernatants with the HIV-1 long terminal repeat (LTR)-luciferase-based reporter cell line TZM-bl (60). We infected TZM-bl cells with a normalized amount of Gag-p24, and after 36 h, we evaluated the activation of the viral LTR. Viruses produced by infected PBT cultivated with bacteria stimulated the HIV-1-LTR 3 to 5 times more than viruses produced by control cells (Fig. 2C). This suggests that viral particles are more infectious and/or that bacteria-conditioned supernatants affect TZM-bl sensitivity to viral particles. The fusogenicity of the viruses generated in the presence of bacteria was thus evaluated using the BlaM-Vpr assay (61). We transfected 293T to produce BlaM-Vpr NL4-3 and NL-AD8 viruses and left 293T cells either untreated or exposed to bacteria during viral production. Bacteria had no effect on the release of viral particles (Fig. 2D). Viral supernatants were ultracentrifuged, and activated PBT were used as target cells. We incubated PBT and viral particles for 2 h, and the presence of BlaM-Vpr in the cytoplasm resulting from virus-to-cell fusion was monitored by flow cytometry. The fusogenicity of NL4-3 or NL-AD8 produced in the presence of bacteria increased 2- to 4-fold (Fig. 2E and F).

We then investigated how the presence of bacteria may affect target cells. PBT were left untreated or cultured with bacteria for 24 h before being used as targets for the BlaM-Vpr fusion assay. We added different doses of NL4-3-BlaM-Vpr viruses produced in the absence of bacteria to target cells (Fig. 2G) and evaluated the ratio between the number of fusion-positive targets and the amount of Gag p24 loaded into cells (Fig. 2H). Cells exposed to bacteria were significantly more likely to fuse than control cells. Thus, bacteria promote cell-free viral fusogenicity and render target cells more prone to fusion with viral particles.

We then wondered if we could detect the effect of bacteria on cell-free viral particles during continuous viral replication. We previously showed that the contribution of cell-free virus-mediated infection may be measured by gently agitating the culture of infected cells (4). In these settings, most cell-to-cell connections are eliminated, and those that can still form are shorter-lived (4). We therefore infected target cells with NL4-3 and established either static or shaking cultures in the presence or absence of bacteria, evaluating the appearance of Gag^+^ cells over time by flow cytometry (Fig. 2I). As expected, in the absence of cell-to-cell contact, viral replication proceeded less efficiently, with 10-fold-fewer Gag^+^ cells present at the peak of viral replication. As previously demonstrated, the presence of bacteria in a static culture increased viral spread. Notably, bacteria also accelerated viral replication in shaken cultures, where the number of Gag^+^ cells doubled compared to the controls (Fig. 2I to L). This suggests that bacteria promote both cell-free and cell-to-cell-mediated infection of PBT.

### Bacterial strains differentially promote T cell activation and modulate T cell markers.

We then examined whether bacteria, in addition to enhancing the infectivity of the viral particles, could modulate expression levels of entry receptors and the activation state of target cells.

**FIG 3:**
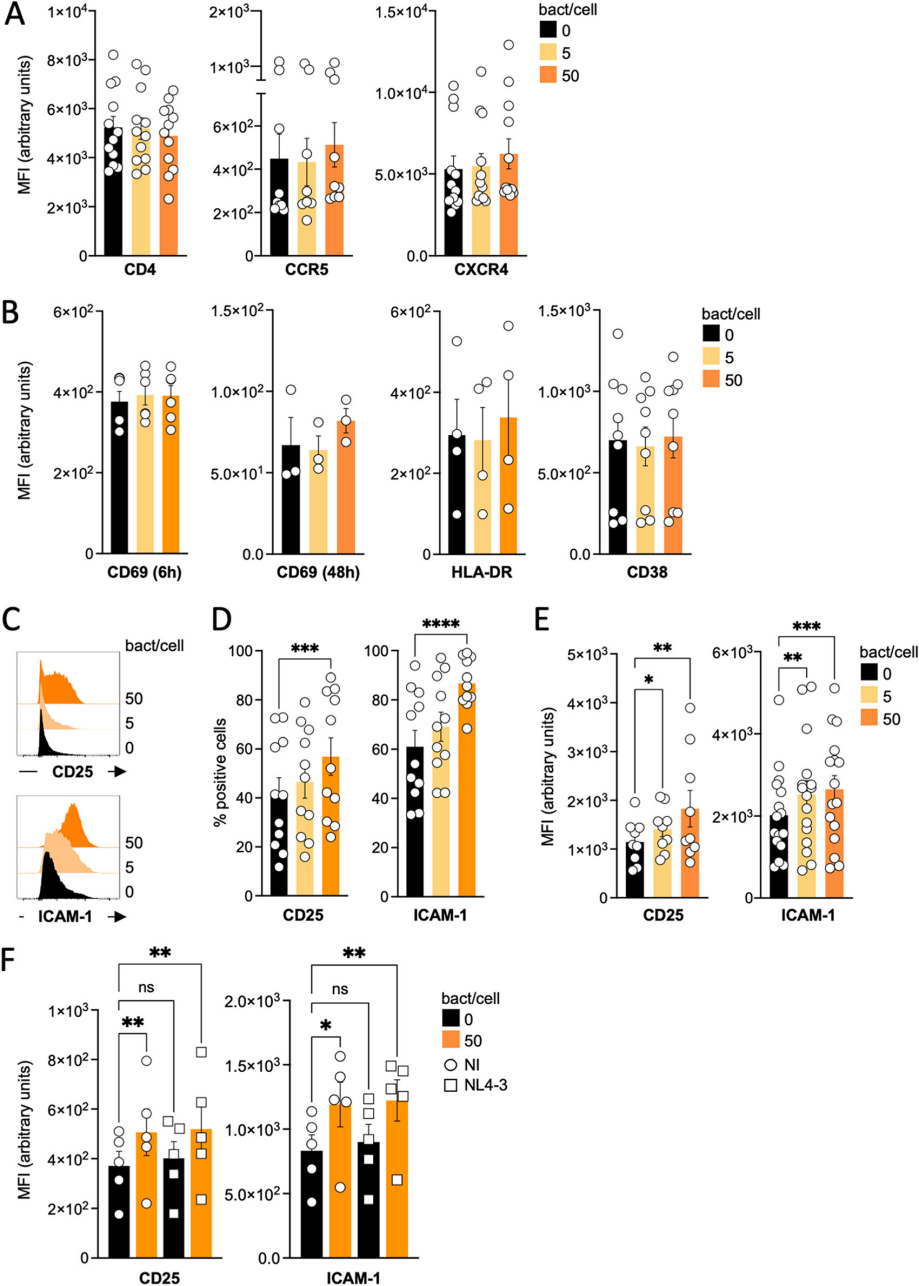
E. coli MG1655 differentially modulates T cell surface molecules. Uninfected activated primary CD4^+^ T cells were set in culture alone or in the presence of 5 or 50 E. coli bacterial cells for 48 h. Cells were then collected, washed, and stained for CD4, CCR5, and CXCR4 (A); CD69 (after 6 or 48 h of coculture), HLA-DR, and CD38 (B); or CD25 and ICAM-1 (C to E) and analyzed by flow cytometry. The mean fluorescence intensity for each cell surface molecule was then calculated for the live cell population. In panel C, a representative analysis of CD25 and ICAM-1 is depicted. (F) NL4-3-infected and uninfected cells were stained to evaluate CD25 and ICAM-1 expression. One out of two experiments is shown. Each histogram shown in panels A, B, and E represents the mean ± SEM of the MFI, calculated for each donor. The histograms shown in panel D represent the mean ± SEM of the percentage of CD25- or ICAM-1-positive cells. Each dot corresponds to an independent donor. The cells of each donor have been left untreated or exposed to the bacteria. Statistical differences between the different conditions have been calculated using the ANOVA paired statistical test. *, *P* ≤ 0.05; **, *P* ≤ 0.01; ***, *P* ≤ 0.001; ns, not significant.

We first established cocultures of E. coli MG1655 bacteria and PBT and assessed surface expression levels of CD4, CCR5, and CXCR4 by flow cytometry, with untreated T cells as control. As shown in Fig. 3A, neither CD4, CCR5, nor CXCR4 was modulated by bacteria. We then analyzed the expression of the same molecules in cells exposed to the Gram-negative or Gram-positive bacteria previously tested for viral replication. As shown in Fig. S2, A. baumannii and P. aeruginosa induced a slight decrease in CCR5 expression, while S. aureus USA300LAC slightly induced CXCR4 expression. However, none of these changes were significantly different from the controls. To evaluate the activation state of the PBT, we started with an exploratory analysis and analyzed some of the cellular markers related to T cell activation, CD69, CD38, HLA-DR, and CD25. We also evaluated the expression of ICAM-1, which has a role both in the infectivity of cell-free viral particles and during cell-to-cell viral transmission (62–64). We incubated PBT with or without E. coli MG1655 bacteria and, after 48 h, analyzed the cells by flow cytometry. The expression of CD69 on untreated cells was assessed at 6 or 48 h after the start of the coculture. As expected, CD69 was barely detectable because the T cells had already been activated for a few days. The presence of bacteria did not affect CD69 expression at either the 6- or 48-h time point. CD38 and HLA-DR were evaluated at 48 h after the start of the coculture. Both molecules were readily detectable, and the presence of bacteria did not affect their levels (Fig. 3B). On the contrary, CD25 and ICAM-1 were consistently upregulated in the presence of E. coli MG1655 bacteria (Fig. 3C to E). We then performed an experiment to determine the minimum ratio of bacteria to cells required to induce the expression of CD25 and ICAM-1 on the cell surface. We started with a ratio of one bacterium per cell and found that this resulted in a consistent and significant induction of both CD25 and ICAM-1 (Fig. S2). As shown in Fig. 3C to E, in the presence of the bacteria, the percentage of cells positive for either marker increased in a dose-dependent manner, and the mean fluorescence intensity (MFI) of the entire live cell population also increased significantly. CD25 and ICAM-1 were also upregulated when the T cells were previously infected (Fig. 3F).

We then tested the ability of our panel of Gram-negative and Gram-positive bacteria to induce CD25 and ICAM-1 expression (Fig. S2). All tested Gram-negative bacteria upregulated ICAM-1. CD25 expression was significantly induced by E. coli Keio and A. baumannii. K. pneumonia, E. cloacae *and* P. aeruginosa did not change CD25’s MFI. Among Gram-positive bacteria, only S. aureus USA300LAC and 132 upregulated CD25 and ICAM-1. Bacteria also induced CD25 and ICAM-1 expression in mobile lymphocytes (Fig. S2). We observed a significant decrease in CD25 and ICAM-1 levels in cells cultured with gentle agitation compared to cells under static conditions (Fig. S2). Thus, bacteria do not promote viral replication by modulating the levels of HIV-1 receptors and coreceptors at the T cells’ surface. Further, they activate T cells and differentially modulate activation markers.

### Bacterial strains differentially promote NF-κB nuclear translocation.

NF-κB activation is important for both viral replication and T cell activation (65, 66). We thus analyzed the activation of PBT using the extent of nuclear NF-κB localization as a readout.

**FIG 4:**
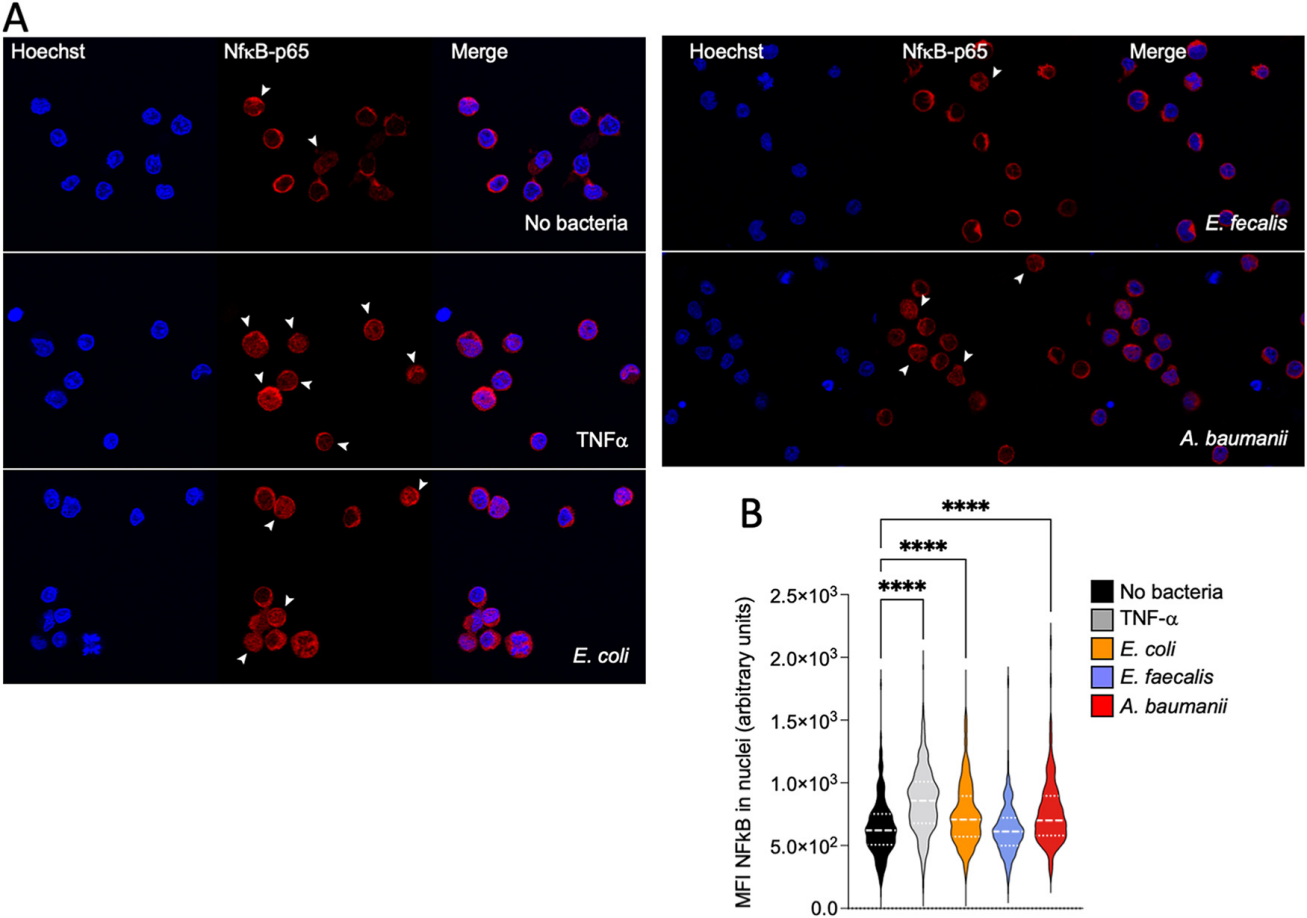
Bacteria differently promote NF-κB nuclear translocation in primary activated CD4^+^ T cells. Uninfected activated primary CD4^+^ T cells were set in culture alone or in the presence of the indicated bacteria for 48 h. As a positive control, cells were also treated with TNF-α. Cells were then collected, washed, and left adhered on a poly-lysine-coated coverslip. After fixation, cells were permeabilized and stained to detect the intracellular localization of NF-κB-p65. Nuclei were stained with Hoechst. Coverslips were then analyzed by confocal microscopy, and single slices were imaged in the central section of the z-stack. (A) Representative images for each condition. The white arrows point to the nuclei that were considered positive for the nuclear translocation of NF-κB. (B) Violin plots showing the distribution of the MFI associated with NF-κB translocated in the nuclei under the different conditions. Between 200 and 600 cells were scored for each condition, derived from 3 to 8 independent donors. Statistical differences between the different conditions have been calculated using the ANOVA unpaired statistical test (Kruskal-Wallis followed by Dunn’s multicomparison test). ****, *P* ≤ 0.0001.

As a positive control, we treated PBT with tumor necrosis factor alpha (TNF-α), which triggers NF-κB translocation in the nucleus within hours. PBT were treated either with 100 ng/mL of TNF-α (as positive control [67]), 50 bacteria/cell, or left untreated. Forty-eight hours after, cells were seeded on poly-lysine-coated coverslips, and the localization of NF-κB-p65 was analyzed by immunofluorescence and confocal microscopy (Fig. 4A). In untreated cells, most of the cells showed a low nuclear localization of NF-κB. TNF-α induced NF-κB activation, as shown by the significant increase of the NF-κB’s MFI associated with the cell nuclei (Fig. 4B). NF-κB nuclear localization was also significantly increased by E. coli MG1655 and A. baumannii, and not by E. faecalis. Thus, bacteria that promote viral replication and CD25 and ICAM-1 upregulation also promote NF-κB nuclear translocation.

### HIV-1 replication correlates with CD25 and ICAM-1 upregulation.

**FIG 5:**
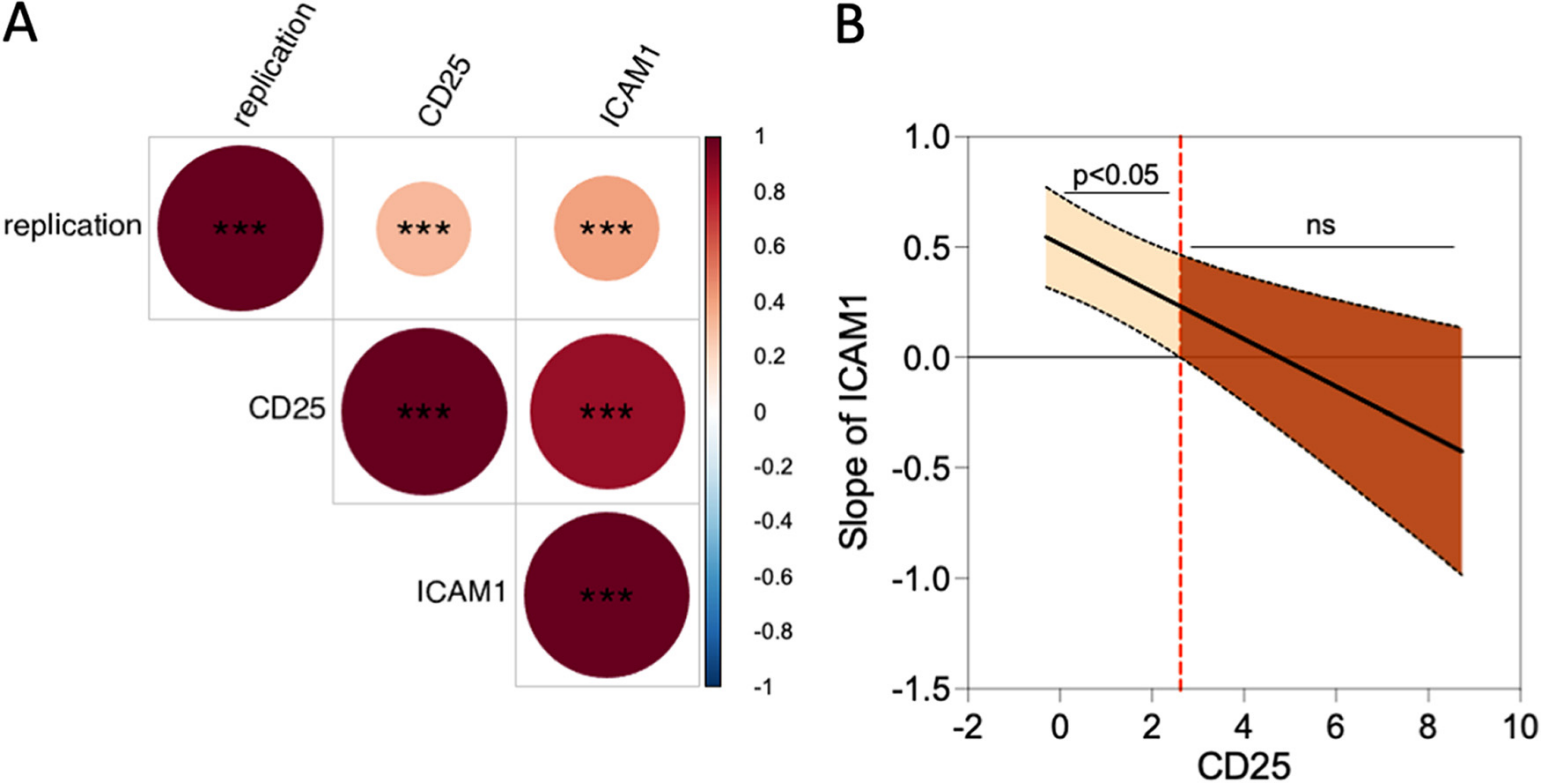
CD25 and ICAM-1 expression correlates with the extent of viral replication in primary CD4^+^ T cells. (A) Pearson correlation matrix of normalized HIV-1 replication and CD25 and ICAM-1 expression. The number of stars indicates the significance of the correlation; ***, *P* ≤ 0.001. Positive correlations are depicted in red. A darker color and a wider circle are associated with a higher correlation coefficient. (B) Impact of ICAM-1-normalized expression on HIV-1-normalized replication depending on the normalized expression of CD25. A simple slope analysis was performed, and Johnson-Neyman intervals were calculated with R as detailed in the Materials and Methods. The slope is significantly different from 0 for values of CD25 expression above the threshold visualized by the dashed line. The 95% confidence intervals are plotted in dashed lines.

The bacteria able to stimulate viral replication were the same ones able to simultaneously upregulate CD25 and ICAM-1 (Fig. 1 and Fig. S2). The correlation between the increase in viral replication and the upregulation of CD25 and ICAM-1 was therefore analyzed. We observed a positive and significant correlation between the effect of bacteria on HIV-1 replication and that on PBT activation (Fig. 5A and Fig. S3). There was also a significant correlation between the extent of upregulation of CD25 and ICAM-1. We analyzed the three parameters together using linear regressions of the replication and simple slope analysis with Johnson-Neyman intervals. We first compared the Bayesian information criterion (BIC) of the model with (25.7) or without (30.7) interaction. The model with interaction had a lower BIC and was thus chosen. We then used simple slope analysis with Johnson-Neyman intervals to show the effect of ICAM-1 expression on replication for a given value of CD25 fold change. The analysis suggested that the upregulation of ICAM-1 had a significant and positive impact on viral replication when the upregulation of CD25 was inferior to 2.6-fold. On the contrary, the role of ICAM-1 on viral replication was minor when CD25 was strongly upregulated. Thus, our analysis suggests that CD25 plays a role during viral replication.

### Bacteria secrete soluble factors stimulating CD25 and ICAM-1 expression on T cells.

**FIG 6:**
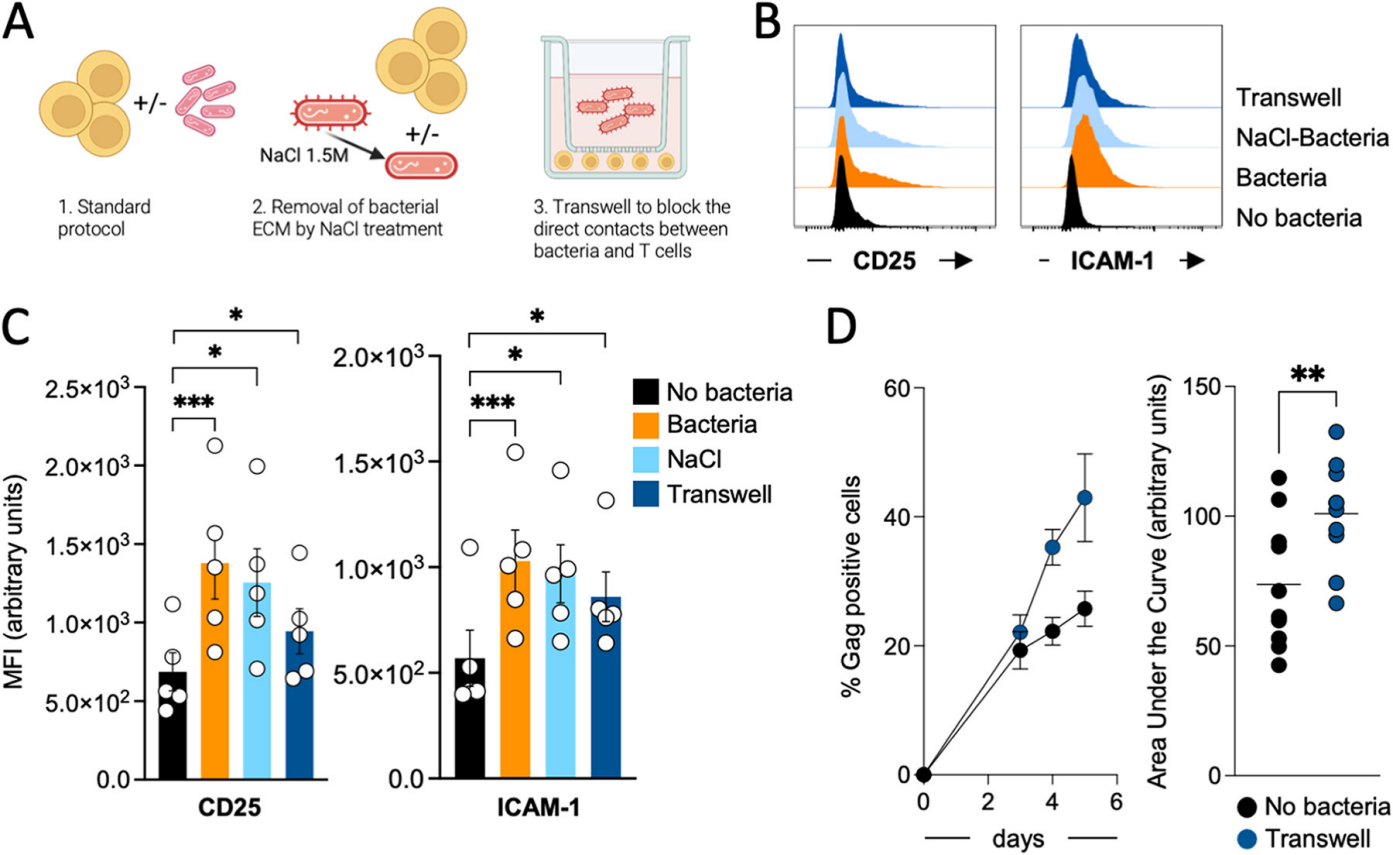
Bacteria secrete factors that promote CD25 and ICAM-1 upregulation as well as viral replication. (A) Schematic overview of the experimental procedure used to determine if bacterial factors promoting T cell activation and viral replication are soluble. (B) Representative histogram overlay analysis of the cell surface expression of CD25 and ICAM-1 under the different conditions. (C) Histograms represent the mean ± SEM of the MFI for CD25 or ICAM-1 in all the donors tested in the various conditions. Each dot corresponds to an independent donor. Cells of each donor have been left untreated or exposed to the bacteria. Statistical differences between the different conditions have been calculated using the ANOVA paired statistical test. *, *P* ≤ 0.05; ***, *P* ≤ 0.001. (D) Replication of the virus in infected primary CD4^+^ T cells that were not treated or cocultivated with bacteria in a Transwell (A, panel 3). Viral replication was followed over a week by flow cytometry. The graph on the left represents the mean ± SEM of the viral replication in a representative experiment. The plot on the right represents the area under the curve of the viral replication over a week under the different conditions. Each dot corresponds to an independent donor. Cells of each donor have been left untreated or cocultivated with bacteria in the Transwell. Statistical differences between the different conditions have been calculated using the Wilcoxon paired *t* test. **, *P* ≤ 0.01.

Next, we investigated whether PBT stimulation required direct bacterial contact. E. coli MG1655 and PBT were cocultivated in various conditions (see experimental outline in Fig. 6A). We used the previously described static coculture protocol as control (Fig. 6A, panel 1). When bacteria divide, they produce a large amount of extracellular material (ECM). ECM contains bacterial proteins, nucleic acids, sugars, and dead cell debris. ECM is bound to bacteria or released into supernatants. Thus, some of these components could act as PAMPs and stimulate PBT, resulting in the observed effects on NF-κB, CD25, and ICAM-1 levels. Before starting the coculture with PBT, we applied a NaCl-based protocol to remove biofilms and strip the ECM from bacteria (Fig. 6A, panel 2) (68). We separated bacteria and PBT using a Transwell filter with a pore size of 0.4 μm (Fig. 6A, panel 3). After 48 h, we evaluated CD25 and ICAM-1 expression on PBT (Fig. 6B and C). NaCl-treated bacteria stimulated PBT as efficiently as the control, suggesting that the ECM did not play a major role. When bacteria and PBT were physically separated by the Transwell, the upregulation of CD25 and ICAM-1 remained significantly higher than in untreated PBT, indicating that some active bacterial factors are released in the supernatants. However, CD25 and ICAM-1 upregulation was less marked than in control cells, suggesting that direct bacterium-cell contacts may further enhance PBT stimulation. When we tested viral replication in PBT maintained in the Transwell, bacteria could still significantly enhance viral spread (Fig. 6D). To confirm that some bacterial factors may be secreted, we filtered the supernatants derived from the E. coli-PBT cocultures and added them to fresh PBT. The supernatants induced CD25 and ICAM-1 upregulation (Fig. S4). We also analyzed the effect of supernatants from bacteria grown without antibiotics. We filtered the bacteria-conditioned medium and added it to new PBT. CD25 and ICAM-1 were both upregulated, thus excluding that the bacterial factors were secreted in response to the antibiotics. We next tested the effect of heat-inactivated bacteria on PBT. We incubated bacteria at 56°C for 1 h (51) before the coculture with PBT. Heat-inactivated bacteria lost nearly all their capacity to stimulate CD25 expression and did not induce ICAM-1 (Fig. S4). Our results therefore demonstrated that bacteria secrete active soluble factors inducing PBT activation and viral replication.

### TLR1, -2, -4, and -5 agonists do not stimulate viral replication and CD25 or ICAM-1 expression.

Considering the well-established interactions between conserved microbial structures called PAMPs and the pattern recognition receptors expressed by immune cells, we examined the impact of various bacterial products and TLR agonists on PBT activation. Uninfected PBT were treated with lipopolysaccharide (LPS) (1 mg/mL), Pam3Cys (100 ng or 1 mg/mL), or heat-killed L. monocytogenes (HKLM; 5 or 50 bacteria/cell) to trigger TLR4, the heterodimer TLR1/2, and TLR2, respectively. Except for the highest dose of Pam3Cys, none of the agonists stimulated CD25, ICAM-1 expression, or viral replication (Fig. S5). Pam3Cys has been shown to be less effective than the purified bacterial Braun lipoprotein to stimulate TLR2 (69). We thus investigated the role of the stimulation of TLR2 by treating PBT with an anti-TLR2 blocking antibody, 50 E. coli MG1655 bacteria/cell, or a combination of the anti-TLR2 antibody and the bacteria. Moreover, we tested two doses of flagellin to activate the TLR5 pathway. The anti-TLR2 antibody alone did not stimulate CD25 or ICAM-1 expression, nor did it inhibit bacterial cells’ effect on PBT activation. TLR5 activation also had no impact (Fig. S5). PBT were also treated with E. coli- or S. aureus-derived peptidoglycans (PGN). None of the two PGN simultaneously induced CD25 and ICAM-1 expression (Fig. S5).

These results showed that the stimulation of various pattern recognition receptors pathways did not recapitulate the effect of bacteria and bacterial supernatants.

### CD25 polarizes at the virological synapse together with HIV-1 Gag.

**FIG 7:**
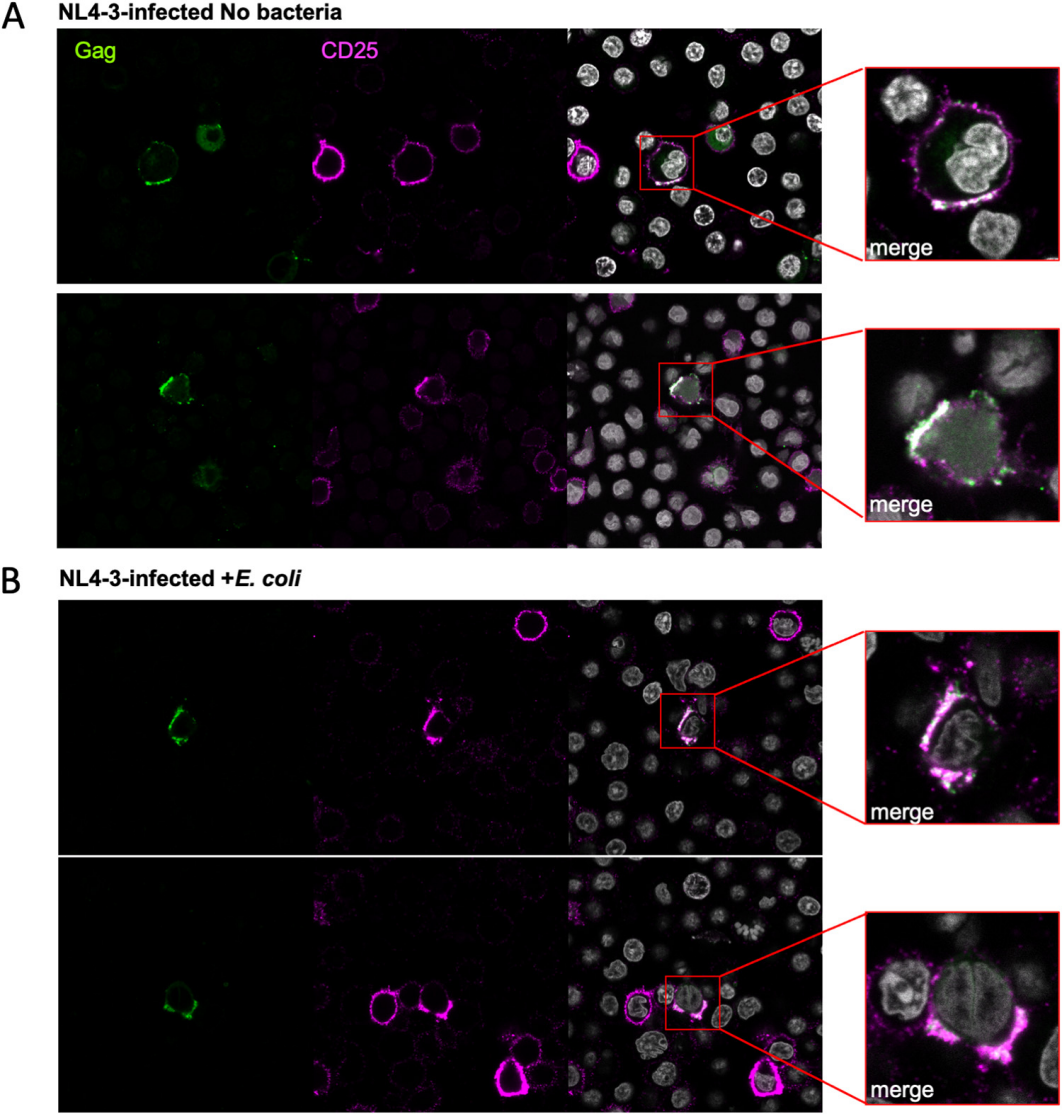
CD25 colocalizes at the virological synapse with HIV-1 Gag proteins. Primary activated CD4^+^ T cells were infected with the NL4-3 virus and cultivated alone or in the presence of E. coli cells. Cells infected for 4 to 5 days were collected, washed, and left to adhere to poly-lysine-coated coverslips. Cells were surface-stained to detect CD25 (purple) and then permeabilized to detect HIV-1 Gag proteins (green). Cells were analyzed by confocal microscopy, and single slices were imaged in the central section of the z-stack. Cell contacts where Gag proteins were accumulated at the interface between one donor-infected cell and one or more uninfected targets were considered virological synapses and analyzed. (A) Representative images of the virological synapses observed in untreated infected T cells. (B) Representative images of the virological synapses observed in the infected cells cocultivated with E. coli. On the right in panels A and B are magnifications of the viral synapses. The white color at the cell surface indicates the area of colocalization between the staining for CD25 and the staining for HIV-1 Gag (merge).

Our findings suggested that CD25 upregulation might play a role in promoting HIV-1 replication (Fig. 5). Interestingly, human T cell leukemia 1 (HTLV-1) spreads via cell-to-cell transmission, and HTLV-1-infected cells upregulate CD25 expression (70–72). Moreover, it has been shown that CD25 triggering in HTLV-1-infected cells induced the microtubule organizing center polarization required to efficiently establish the cell-to-cell contacts and viral transmission (73). Since cell-to-cell transmission accounts for most of the viral spread in our experimental settings, we hypothesized that in infected PBT exposed to bacteria, CD25 could have a role at the VS, as in HTLV-1 infected cells. To explore our hypothesis, we infected PBT and cultivated them with or without E. coli MG1655. Target cells were also left untreated or maintained with bacteria. After 3 days, productively infected cells were mixed for 3 h (1:1 ratio) with targets to allow VS formation. After fixation, PBT were stained to detect surface levels of CD25 and permeabilized to visualize intracellular HIV-1 Gag proteins. VS were defined as cell-cell contacts where HIV-1 Gag polarized at the zone between donor and target cells. Because they were activated for 5 to 7 days before the coculture, not all PBT stained positive for CD25. We first focused on the VS formed by donor cells that stained double positive for Gag and CD25. CD25 was found to colocalize with Gag at the VS (Fig. 7A and B and Fig. S6). CD25 and Gag colocalization was observed in cocultures of T cells exposed or not to bacteria. Infected cells that did not express detectable levels of CD25 still formed VS with target cells (Fig. S6). We could not find infected cells with CD25 polarized at cell-cell contacts without Gag.

Taken together, our results suggest that CD25 is not required to form VS, but when PBT expressed CD25, it polarizes at the VS together with HIV-1 Gag. Further, CD25 is recruited at the VS irrespective of the presence of bacteria. Bacteria can increase the number of VS in which CD25 is present.

## DISCUSSION

In this work, we show that several Gram-negative and Gram-positive bacteria stimulate HIV-1 replication in PBT. Increased viral replication correlates with the upregulation of CD25 and ICAM-1. Viral replication and PBT activation are stimulated by bacterial soluble factors. We further demonstrate that CD25 localizes at the VS with Gag.

HIV-1 infection is characterized by a progressive depletion of circulating CD4^+^ T cells and by the establishment of a chronic immune activation state marked by a high turnover of T cells, the activation of B cells and T cells, and elevated levels of proinflammatory mediators. While these responses may be helpful or even required during acute infection, they may contribute to the worsening of the disease during the chronic phases (43). Microbial translocation plays a significant role in maintaining immunological activation in people living with HIV-1 (31, 74). Peptidoglycans, lipopolysaccharide (LPS), flagellin, and ribosomal RNA genes (rRNA genes) are examples of bacterial products released following the disruption of the gastrointestinal barrier, but others, yet to be identified, are likely also released.

Microbial compounds are present in the bloodstream and in distant lymph nodes (27, 28), and circulating T cells are continuously exposed to them. Our results now show that circulating T cells react to the presence of certain bacteria *in vitro* and support higher levels of viral replication of both R5 and X4 HIV-1 strains. The stimulation of T cells by circulating microbial products may facilitate the onset of infection from the early stages and subsequently contribute to the maintenance of chronic immune activation during the later stages of infection. Whether one of these effects may have a more pronounced impact on the course of the infection remains to be further investigated. The viruses relying on CCR5 for entry were slightly less sensitive to bacteria, whether that dependence was partial (SF2) or total (NL-AD8). One interesting possibility would be that X4-tropic viruses are selected as the disease develops because of their increased susceptibility to the effects of bacterial products. Future work would be required to test this hypothesis, for instance, by testing a panel of transmitted founder viruses in combination with their X4 variants isolated later in the infection.

This work extends previous observations by Dillon and colleagues who demonstrated increased HIV-1 replication and cell activation in lamina propria T cells (LPT) exposed to E. coli (51). We now show that various bacteria can activate PBT as well and demonstrate that this occurs via the secretion of soluble bacterial factors. Dillon et al. found no evidence of increased viral replication in total peripheral blood mononuclear cells (PBMCs) after exposure to E. coli and concluded that the ability of E. coli to promote viral replication was limited to LPT (51). Yet, whereas in total PBMCs, the T cells are in a quiescent state that, by itself, does not allow viral replication, we used activated PBT, which support high levels of viral replication. Moreover, PBMCs were exposed to heat-inactivated bacteria, which cannot secrete bacterial factors and also, in our hands, were unable to further activate PBT. Therefore, these experimental differences likely explain our different conclusions. It will be interesting, however, to test the response of resting PBT to bacteria which have not been heat inactivated, both in infectious and noninfectious settings. Further, LPT have a basal activation level that is higher than PBT (17). This could explain why they were able to react to heat-inactivated bacteria. Another intriguing hypothesis would be that, depending on the T cell type, different bacteria and/or bacterial factors promote T cell activation and viral replication.

Bacteria increased the expression of CD25 and ICAM-1, both in uninfected and in infected cells, but had no effect on other activation markers (such as HLA-DR or CD38) and on CD4, CCR5, or CXCR4. We anticipate that additional cellular markers could be affected by the presence of bacteria, and future research will allow us to identify them. ICAM-1 upregulation, following bacteria exposure, has been previously reported in other cell types (75, 76). We now demonstrate that several Gram-negative bacteria increased ICAM-1 expression on PBT, while only the two MRSA strains were able to do so among the Gram-positive bacteria tested in our panel. In contrast, only the Gram-negative E. coli and A. baumannii and the two MRSA strains significantly upregulated CD25.

We show that bacteria stimulate PBT activation and viral replication by releasing soluble products in the culture. However, as in the absence of bacterium-PBT contacts, the upregulation of CD25 and ICAM-1 was slightly less efficient (Fig. 6); it is possible that a direct contact between bacteria and T cells further promotes PBT activation. Alternatively, the higher local concentration of bacterial factors that is expected in the coculture compared to the Transwell condition could also explain this difference. The bacterial factors that stimulate PBTs are soluble, rendering our observations more pertinent from a physiological standpoint. In fact, as shown for LPS and other products (27, 77), these factors may enter the bloodstream and peripheral lymph nodes and thus be able to encounter infected or uninfected PBTs. Our results suggest that the bacterial factors stimulating ICAM-1 and CD25 expression are not the same, as one would otherwise expect all Gram-negative bacteria to upregulate CD25 as well. Among the TLR agonists we tested, only Pam3Cys slightly stimulated PBT and viral replication (Fig. S5). However, when we used an anti-TLR2 blocking antibody, we were not able to block PBT activation by E. coli. Thus, further investigations are warranted to identify both the bacterial products and cellular pathways stimulating PBT activation and viral replication.

Although we have yet to identify the molecular mechanisms underlying the link between bacteria-mediated PBT activation and increased viral replication, we show that HIV-1 replication is increased by the bacteria that simultaneously upregulated ICAM-1 and CD25, but not from those that only upregulated ICAM-1. This was unexpected given that ICAM-1 is incorporated into viral particles, favors HIV-1 infectivity and entry into target cells (12, 64, 78–80), stabilizes the VS, and allows more efficient viral transmission (10). However, our data show that cell-free viral particles produced in the presence of bacteria are more infectious and that bacteria enhance viral spread in mobile lymphocytes, where cell-to-cell contacts are strongly reduced. Thus, we are tempted to speculate that bacteria-mediated ICAM-1 upregulation could contribute to enhanced viral replication by promoting cell-free HIV-1 infectivity.

Our findings suggested that high expression of CD25 alone may be sufficient to promote viral replication (Fig. 5). This result prompted us to investigate whether CD25 could be present at the VS, which is not only the main transmission mode in our experimental conditions but also the most efficient. We show, for the first time, that CD25 colocalizes with Gag at the VS, both in the presence of bacteria and in untreated cells, suggesting that CD25 polarization does not require the bacterial stimulus to occur. However, we expect that PBT exposed to bacteria would form a higher number of CD25-positive VS than untreated PBT and that the amount of CD25 localized at the synapse would also be higher. Our data show that Gag does not require CD25 to accumulate at the VS, which is consistent with data showing VS formation in CD25-negative cell lines. Interestingly, CD25 expression combined with other activation markers has been shown to predict HIV-1 permissiveness of CD4^+^ T cells (81, 82). Further, regulatory T cells (Tregs), expressing high levels of CD25, have been shown to be susceptible to HIV-1 infection (82–84). Further analyses will elucidate if bacteria-induced CD25-high PBT have also a Treg phenotype.

CD25 polarization has been described during homotypic T-T cell interactions, and it has been proposed that the polarization of CD25 would increase the local amount of interleukin 2 (IL-2) and sustains cell proliferation by promoting STAT5 signaling cascade in adjacent cells during T cell proliferation (85). VS can be considered a peculiar case of homotypic T-T cell interactions. Thus, it will be interesting to investigate if there is a distinct target infection outcome depending on whether CD25 is present at the VS. If CD25 plays a role in cell-free virus infection also remains to be investigated. It is worth noting that the extent of CD25 expression in T cells activated *in vitro* versus *in vivo* can differ significantly (86). Thus, it will be important to confirm these findings through further research, such as the use of humanized mice infected with HIV-1.

Our findings reveal a complex interaction among bacteria, T cells, and HIV-1, where bacteria can affect T cell activation, which, in turn, leads to increased viral replication. This type of interaction is not unique to HIV-1, as functional interplays between viruses and bacteria have been previously described. For example, it has been reported that enteroviruses can attach to the surface of gut bacteria and promote infection, facilitating viral coinfection of target cells and promoting viral gene recombination (87). It was also shown that treatment with antibiotics, which reduces the number of bacteria in the intestine, significantly reduces poliovirus infection (88). Furthermore, mouse mammary tumor virus (MMTV) is a retrovirus that preferentially infects via mucosal membranes. Kane and colleagues showed how the presence of the mucosa-associated microbiota is necessary for MMTV transmission (89). Interestingly, respiratory viruses have been reported to promote bacterial spread (reviewed in reference 90). Thus, the ability of these microorganisms to boost each other's dissemination can occur in both directions.

Future studies on the links between bacteria, viruses, and cells will help elucidate how these interactions contribute to the development of diseases.

## MATERIALS AND METHODS

### Bacterial strains.

Bacterial strains used in this study are listed in Table S1 in the supplemental material. Experiments were performed in lysogeny broth (LB) or RPMI medium enriched with 10% fetal bovine serum (FBS). Culture media have been supplemented or not with penicillin-streptomycin (Pen-Strep) mix (200 U/mL; Thermo Fisher; catalog no. 15140122).

### HIV-1 Gag and T cell marker staining.

To evaluate the expression of T cell markers, we collected cells from culture and washed them in phosphate-buffered saline (PBS). Cells were then colored with the following antibodies diluted in PBS, 1% BSA, and 0.01% Na azide (staining buffer): anti-human CD4-phycoerythrin (PE) (ImmunoTools; clone MEM-241; 1:20 dilution); CCR5-Alexa647 (BioLegend; catalog no. 313712; 1:100 dilution); CXCR4-allophycocyanin (APC) (BD Biosciences; catalog no. 555976; 1:50 dilution); HLA-DR-PE (Miltenyi; catalog no. 130-096-177; 1:100 dilution); CD69-PE (Immunotools; 1:20 dilution); CD38-PE (BD Biosciences; catalog no. 555460; 1:50 dilution); CD25-PE or -APC (ImmunoTools; clone MEM-181 or clone HI25a; 1:20 dilution); and CD54-ICAM-1-PE or APC (ImmunoTools; clone 1H4; 1:20 dilution). After staining for 30 min at 4°C, cells were washed and fixed in 4% paraformaldehyde (PFA) before being analyzed by flow cytometry. To stain HIV-1 Gag proteins in HIV-infected T cells, cells were first fixed for 10 min in 4% PFA and then washed in PBS. Beckman Coulter's anti-HIV-1 Gag antibody (clone KC-57-rhodamine or fluorescein isothiocyanate; 1:500 dilution) was diluted in staining buffer with 0.05% saponin (Sigma) and then used to stain cells for 30 min. Cells were then washed and analyzed by flow cytometry. To acquire samples, we used a BD FACSCanto II, and the results were analyzed by FlowJo 10.6 software.

### Cells.

293T and TZM-bl cells were maintained in culture in Dulbecco's modified Eagle medium (DMEM) (Invitrogen) supplemented with 10% FBS and Pen-Strep. Primary CD4^+^ T cells were purified from human peripheral blood by density gradient centrifugation (lymphocyte separation medium 1077; PAA Laboratories, Merck) followed by immunomagnetic selection (Miltenyi). More than 98% of the cells were CD4^+^ CD3^+^. For activation, T cells were treated either with phytohemagglutinin (PHA) or with anti-CD3/CD28/CD2-coated beads (Miltenyi). To stimulate T cells with PHA, purified CD4^+^ T cells maintained at 5 × 10^6^ million/mL in Roswell Park Memorial Institute (RPMI) medium were treated overnight with PHA (1 μg/mL; Oxoid, Thermo Fisher). The day after, cells were diluted 1:10 in interleukin 2 (IL-2)-containing RPMI (50 IU/mL; R&D Systems) supplemented with 10% FBS and Pen-Strep (RPMI-IL-2) and maintained in culture until use. To activate CD4^+^ T cells with beads, we followed the manufacturer’s instructions and used a bead-to-cell ratio of 1:4. Bead-activated T cells were then cultivated in RPMI-IL-2, as suggested by the manufacturer, until they were used for subsequent manipulations.

### Production of viral particles.

Viral particles were produced by CaCl_2_ transfection of 293T cells. Viruses used to infect target cells were not ultracentrifuged. To produce Blam-Vpr-positive viruses (61) in 293T cells exposed to bacteria, we followed our previously described protocol (91) with some modifications. On the day of the transfection, 293T cells from an extra flask were detached and used to estimate the number of cells present in the flasks. When stated, bacterial cells, prepared as described below for T cells, were added to the cells right after the DNA precipitate. The day after, the medium was changed, and new bacterial cells were added to the culture. Forty-eight hours after transfection, supernatants containing the viral particles were collected and filtered with a 0.45-μm filter to remove cell debris and, when present, bacterial cells. Viral particles were concentrated by ultracentrifugation, and the amount of virus produced was quantified by ELISA following the manufacturer’s instructions. Blam-Vpr viral particles produced in the absence of bacteria were used as controls. Aliquots of the ultracentrifuged viral stocks were stored at −80°C before being used for the experiments.

### Coculture between bacteria and primary CD4^+^ T cells.

A single colony of bacteria was inoculated in lysogenic broth (LB) and grown overnight at 37°C, reaching stationary phase after 8 to 9 h, after which bacteria were collected and centrifuged for 5 min at 2,000 × *g*. LB was removed and pellets were washed once in PBS and then resuspended in PBS in one-quarter of the original culture volume. The bacterial number per milliliter was then estimated by measuring the optical density at 600 nm. To remove the extracellular material (ECM) from the surface of bacteria, we followed the protocol described in reference 68. Briefly, after overnight growth in LB medium, bacteria were collected, centrifuged, and washed once in PBS. After further centrifugation, the bacterial pellet was resuspended in a 1.5-M NaCl solution for 5 min. Bacteria were then centrifuged, and the supernatant containing the ECM was discarded. Bacteria were resuspended in PBS and used for subsequent experiments. Primary CD4^+^ T cells were resuspended in RPMI-IL-2 containing 2× Pen-Strep (CM) at 10^6^ cells/mL. Importantly, to ensure the efficacy of the antibiotics, CM was freshly prepared for each experiment. Bacteria were then added or not at a ratio of 5:1 or 50:1 to primary CD4^+^ T cells. Cells were cultivated in a 24-well plate for 48 h unless otherwise indicated. When Transwells (Corning) were used, bacteria were inoculated in the upper chamber and T cells in the lower.

### Infection of primary CD4^+^ T cells in the presence of bacterial cells.

Primary CD4^+^ T cells were resuspended in CM at 10^6^ cells/mL. Cells were infected with the indicated viral strains (NL4-3 [92], SF2 [93], Bru [94], and NL-AD8 [95]). An equal amount of Gag p24, ranging from 20 to 100 ng/mL, was used, depending on the experiment. Diethylaminomethyl-dextran (DEAE dextran) (1 μg/mL) was added during the infection to promote viral adsorption. Infections were carried out at 37°C with intermittent and gentle agitation in a Thermomixer (Eppendorf) for 3 h. At the end of the infection, unbound viral particles were removed by centrifugation at 1,500 rpm for 5 min, and cells were washed in PBS. Cells were resuspended in fresh CM at 10^6^ cells/mL, and 1 mL/well was distributed in a 24-well plate. Bacterial cells were then added or not to T cells at a ratio of 5:1 or 50:1. Cells were cultivated over a week for subsequent manipulations. To analyze viral replication in the absence of cell contact, we used the protocol described in reference 4. After infecting T cells, we divided the cells into 24-well plates for static cultures and 12-well plates for mobile cultures. The same amount of medium was used for both conditions, and bacteria were added or not to the culture. The cells were left on the rocker for the duration of the experiment, and viral replication under these conditions was compared to that under static conditions.

### Analysis of viral infectivity.

The TZM-bl cell line (originally called JC.53-BL) was generated from the clone JC.53 expressing a high level of CD4 and CCR5 and endogenously expressing CXCR4 (60). This cell line is highly sensitive to infection by most viral strains. TZM-bl cells were infected in triplicate with an equivalent amount of Gag p24 (1 or 10 ng/mL) of the indicated viruses. At 48 h after infection, the relative viral infectivity was measured using a luciferase assay with Bright-Glo (Promega) reagent according to the manufacturer’s instructions.

### Fusion assay.

NL4-3 and NL-AD8 BlaM-Vpr viral particles were produced in 293T as described above and in reference 61. Target-activated CD4^+^ T cells were plated in 96-well plates at 10^6^ cells/mL and 100 μL/well. Viruses were added at the indicated concentrations, ranging from 10 to 500 ng/mL depending on the experiment, and cells were incubated for 2 h at 37°C. After extensive washing in PBS, T cells were loaded with the CCF2-AM loading kit (Invitrogen) in the presence of 1.8 mM probenecid (Sigma). Cells were incubated for 2 h at room temperature in the dark and then washed three times in PBS and fixed using 4% PFA. The cleaved CCF2-AM fluorescence (excitation at 405 nm, emission at 450 nm) was measured by flow cytometry on a FacsCanto II (BD). When indicated, target cells were cultivated with bacteria (50:1 ratio) for 24 h.

### Immunofluorescence and fluorescence analysis.

Primary CD4^+^ T cells were left untreated or exposed to the indicated bacteria for 24 h. Cells were collected, washed from the culture medium, resuspended in PBS, and left to seed onto a 0.01% poly-lysine-coated coverslip for 30 min. Cells were fixed for 10 min in 4% PFA. To allow NF-κB staining, cells were permeabilized for 30 min in 0.5% Triton-X, PBS, and 1% BSA and then left for 30 more minutes in PBS and 1% BSA. Staining was performed using the rabbit anti-human NF-κB antibody (Cell Signaling Technology; catalog no. 8242; diluted 1:500) for 1 h at room temperature. After washing, goat anti-rabbit CY3 (Jackson ImmunoResearch; catalog 111-165-003; diluted 1:200) was used as a secondary antibody and left for 30 min. Nuclei were stained using Hoechst 33342 (Invitrogen; catalog no. H3570; diluted in PBS and 1% BSA for 5 min). Cells were rinsed in PBS and 1% BSA, and, after being briefly washed in distilled water, the coverslip was mounted on glass support using the Prolong Diamond antifade mountant (Invitrogen; catalog no. P36970).

Confocal images were acquired using an LSM700 confocal microscope (Zeiss) with standardized laser settings accordingly to the negative controls (secondary and primary only). For nuclear quantification of NF-κB signal, we used a protocol slightly modified from reference 96. Prior image analysis, raw files were preprocessed using a median filter (2-pixel radius). Binary images representative of the nuclei were obtained by Otsu thresholding (97) and set up as regions of interest (ROIs). The nuclei ROI areas were then used in the raw CY3 slices to measure the intensity of NF-κB signal in each cell by their mean gray intensity. Apoptotic or dividing cell nuclei were manually excluded from the analysis due to aberrant nuclear staining. Image manipulation was conducted using Fiji software. The mean fluorescence intensity for the NF-κB staining inside the nuclei was then quantified in the different conditions, and statistical differences were analyzed using GraphPad Prism 9 software.

### Virological synapse formation and visualization.

Activated T cells were infected with NL4-3 as described before and left in culture untreated or in the presence of E. coli cells at a 50:1 ratio of bacteria to T cells. Three or 4 days after infection, cells were collected and mixed at a 1:1 ratio with uninfected target cells that had also been kept in culture without or with bacteria. Mixed donor and target cells were seeded onto a poly-lysine-coated glass coverslip before being cultured at 37°C for 1 to 2 h to let the virological synapses form. Cells were then washed and fixed in PBS-PFA 1% for 15 min. Cells were first stained with an anti-human CD25 antibody (ImmunoTools; clone MEM-181 or clone HI25a; 1:20 dilution), followed by a secondary antibody (Jackson, goat anti-mouse IgG1-CY3 or -A647 conjugated, depending on the experiment; 1:200 dilution). Cells were then permeabilized in PBS, BSA 1%, and saponin 0.05% and stained with a rabbit anti-HIV-1 Gag-p24 (NIH AIDS reagents; catalog no. 4250; 1:500 dilution), followed by a secondary antibody (Jackson; goat anti-rabbit A488; 1:200 dilution). After staining, nuclei were colored with Hoechst (Invitrogen; catalog no. H3570; 1,10.000 dilution in PBS, 5 min). Coverslips were then put on glass slides with ProLong Diamond antifade mountant (Invitrogen; catalog no. P36970) and left overnight to dry.

Virological synapses were visualized using a confocal microscope (LSM700; Zeiss), and images were processed and analyzed using Fiji and Imaris software.

### Statistical analysis.

Pearson correlation matrix and interactions between HIV-1 replication and ICAM-1 and CD25 expression were calculated with RStudio 2021.09.1 using R version 4.1.0. The following packages were used: readxl (https://CRAN.R-project.org/package=readxl), pheatmap (https://CRAN.R-project.org/package=pheatmap), corrplot (https://github.com/taiyun/corrplot), caret (https://CRAN.R-project.org/package=caret), and interactions (https://CRAN.R-project.org/package=interactions). Expressions and HIV-1 replication data were normalized on the “no bacteria” condition. The Pearson correlation matrix was plotted with the function rquery.cormat from http://www.sthda.com/upload/rquery_cormat.r. To study the interactions between these 3 parameters, we performed 2 linear regressions (one with and one without an interaction term). Data were not following a normal distribution and were then modified with a Box-Cox transformation before the regressions. Bayesian information criterion (BIC) was calculated for each model using the BIC function of the stats package. The simple slope analysis and Johnson-Neyman interval calculation of the linear regression lm(rep_new~ ICAM-1*CD25) was performed with the sim_slopes function using ICAM-1 expression as predictor and CD25 expression as moderator variable.
