# Supplementary material for: Exposure to Secreted Bacterial Factors Promotes HIV-1 Replication in CD4+ T Cells
Source: Microbiol Spectr. 2023 Feb 28;11(2):e04313-22. doi: 10.1128/spectrum.04313-22 (PMC10100953; doi:10.1128/spectrum.04313-22)
Supplement: Supplemental file 1 — Supplemental material. Download spectrum.04313-22-s0001.pdf, PDF file, 5.2 MB [file spectrum.04313-22-s0001.pdf]

S1 Table. List of the bacteria used in this study.

Name	Phylum	Family	Association with people living with HIV - PLWH	Strain references
*E. coli (*Gram-)	Proteobacteria	Enterobacteriaceae	(1-3)	MG1655 (4)<br><br>BW25113 (5)
A. baumanii (Gram-)	Proteobacteria	Moraxellaceae	(6-8)	Acinetobacter_baumanii_ATCC17978
K. pneumoniae LM21 (Gram-)	Proteobacteria	Enterobacteriaceae	(7, 9, 10)	(11)
E. cloacae U28(Gram-)	Proteobacteria	Enterobacteriaceae	Not associated with PLWH	Gift from C. Forestier
P. aeruginosa PAO1 (Gram-)	Proteobacteria	Pseudomonadaceae	(7, 12)	(13)
E. fecalis (Gram+)	Firmicutes	Enterococcaceae	(14)	(15)
B. subtilis 168 (Gram+)	Firmicutes	Bacillaceae	Not associated with PLWH	(16)
S. aureus 15981 (Gram+)	Firmicutes	Staphylococcaceae	(7, 17, 18)	(19)
S. aureus 132 (MRSA Gram+)	Firmicutes	Staphylococcaceae	(7, 17, 18)	(20)
S. aureus USA300 (MRSA Gram+)	Firmicutes	Staphylococcaceae	(7, 17, 18)	(21)

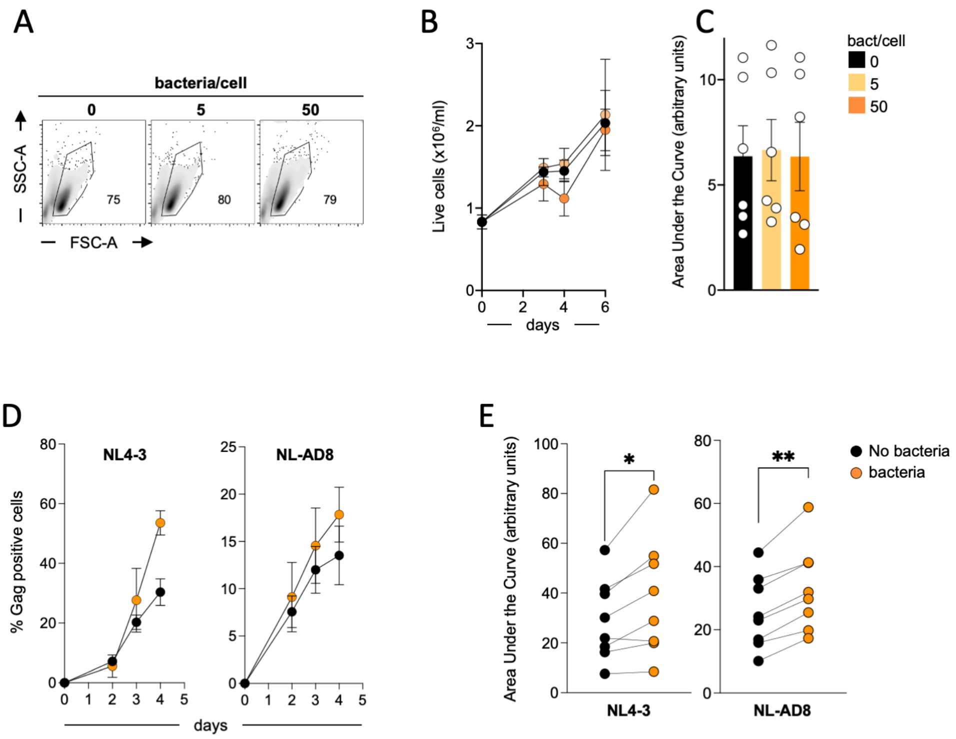

S1 Fig. Bacteria do not affect cell viability and promote viral replication in CD3/CD28/CD2 activated CD4+ T cells. (A-C) The viability of activated primary CD4+ T cells is not affected by *E. coli*. (A) Flow cytometry analysis of the FSC-SSC profile of activated primary CD4+ T cells that were left untreated or co-cultured with 5 or 50 bacteria for three days. The percentage of live cells in the gate is indicated by the numbers in the density plots. (B) Primary CD4+ T cells were either left untreated or cultured in the presence of *E. coli* at the indicated ratios; cells were counted over one week using trypan-blue to detect dead cells. Mean +/- SEM of the number of live cells (B); Mean +/- SEM of the area under the curve for each donor (C). Each dot represents a different donor. Cells of each donor were either left alone or co-cultured with bacteria. (D-E) When co-cultured with *E. coli*, primary CD4+ T cells activated with anti-human CD3, CD28, and CD2-coated beads replicate HIV-1 more efficiently. (D) Flow cytometry was used to track the appearance of Gag+ cells in culture in NL4-3 or NL-AD8-infected primary CD4+ T cells over a week. The graphs show the means+/- SEM of viral replication detected in four donors in a representative experiment. (E) Analysis of the area under the curve of viral replication in untreated or bacteria-exposed T cells. For each infection, the area under the curve has been calculated for each donor. Each dot represents a specific donor. Cells of each donor have either been left untreated or have been exposed to *E. coli* (50 bacteria/cell). Statistical differences between the different conditions have been calculated using the Wilcoxon paired t-test. *=p≤0.05; **=p≤0.01.

S1 Fig.

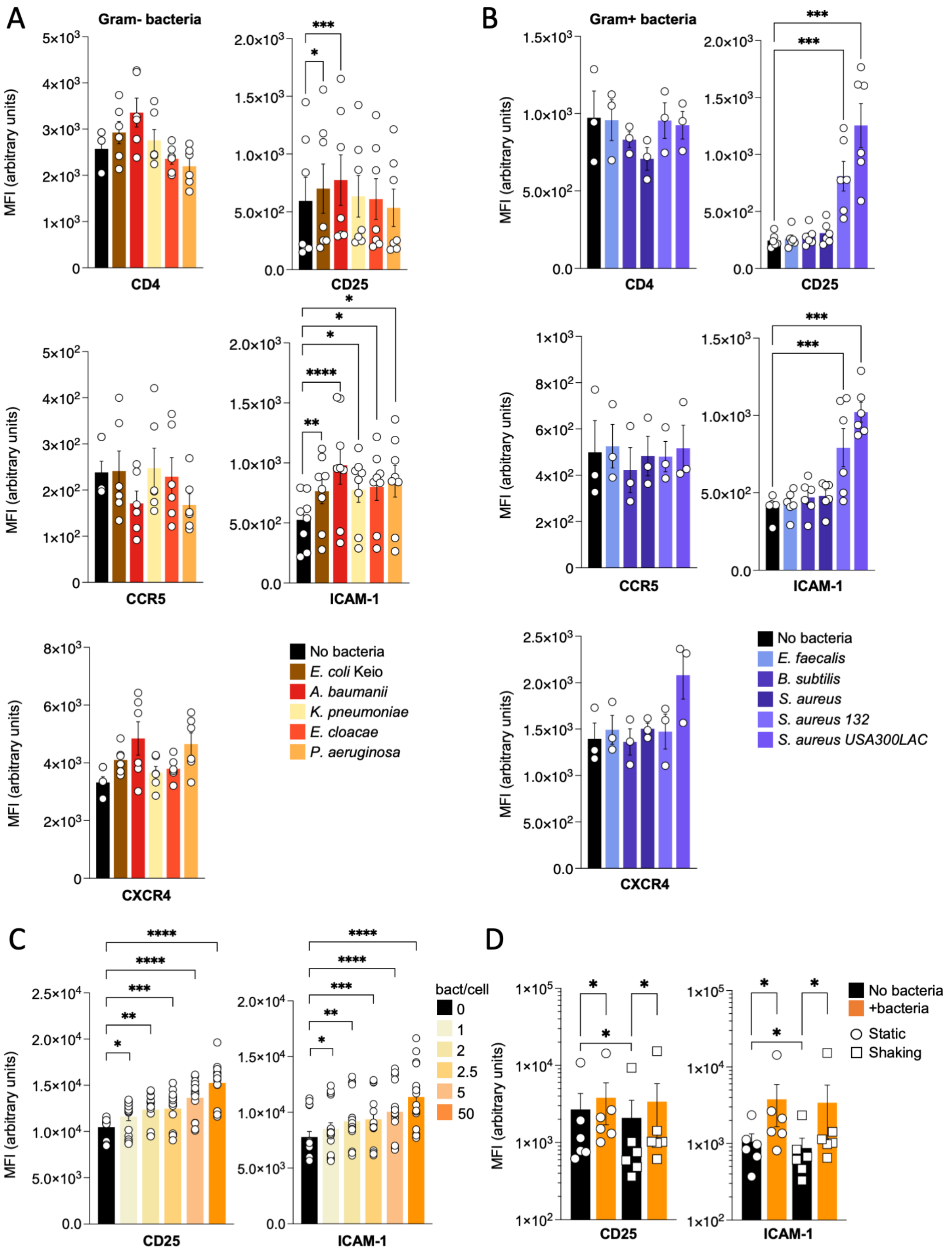
A
Gram- bacteria
MFI (arbitrary units)
CD4
CD25
CCR5
ICAM-1
CXCR4
No bacteria
E. coli Keio
A. baumanii
K. pneumoniae
E. cloacae
P. aeruginosa
B
Gram+ bacteria
CD4
CD25
CCR5
ICAM-1
CXCR4
No bacteria
E. faecalis
B. subtilis
S. aureus
S. aureus 132
S. aureus USA300LAC
C
CD25
ICAM-1
bact/cell
0
1
2
2.5
5
50
D
CD25
ICAM-1
No bacteria
+bacteria
Static
Shaking

S2 Fig.

S2 Fig. Cell surface molecules are modulated by Gram negative (-) and Gram positive (+) bacteria. (A-B) Uninfected activated primary CD4+ T cells were either left untreated or co-cultured with the indicated Gram- (A) or Gram+ (B) bacteria. Cells were collected and stained for the indicated molecules forty-eight hours later. Flow cytometry was used to analyze the cells, and the MFI for each molecule was calculated on the population of live cells. The histograms in (A and B) represent the MFI's mean +/- SEM. Three to eight independent donors were left untreated or exposed to 5 or 50 bacteria/cell, and the data were compiled. Depending on the experimental design, statistical differences between conditions were calculated using either the ANOVA Kruskall-Wallis unpaired test or the ANOVA paired Friedman test. *= $p \leq 0.05$; **=$p \leq 0.01$; ***=$p \leq 0.001$; ****= $p \leq 0.0001$. (C) Uninfected activated primary CD4+ T cells were either exposed to the indicated number of bacteria/cell or left untreated. Forty-eight hours after cells were collected and stained to evaluate by flow cytometry the expression of either CD25 or ICAM-1. The histograms show the mean +/- SEM of the MFI for each molecule. Three independent donors were tested in triplicate. Each dot represents a single well. *= $p \leq 0.05$; **=$p \leq 0.01$; ***=$p \leq 0.001$; ****= $p \leq 0.0001$.(D) *E. coli* regulates the expression of CD25 and ICAM-1 on the cell surface of primary CD4+ T cells cultivated in static and mobile cultures. Uninfected primary CD4+ T cells were cultured in static conditions or with gentle agitation for 48 hours, either alone or in the presence of bacteria. Flow cytometry was used to assess the MFI for CD25 and ICAM-1. The mean +/- SEM of the different donors who were tested is shown. Each symbol represents a different donor. Cells of each donor have been left untreated, exposed to bacteria, cultured in static culture, or agitated gently. The Wilcoxon paired t-test was used to calculate statistical differences between the various conditions. *=$p \leq 0.05$.

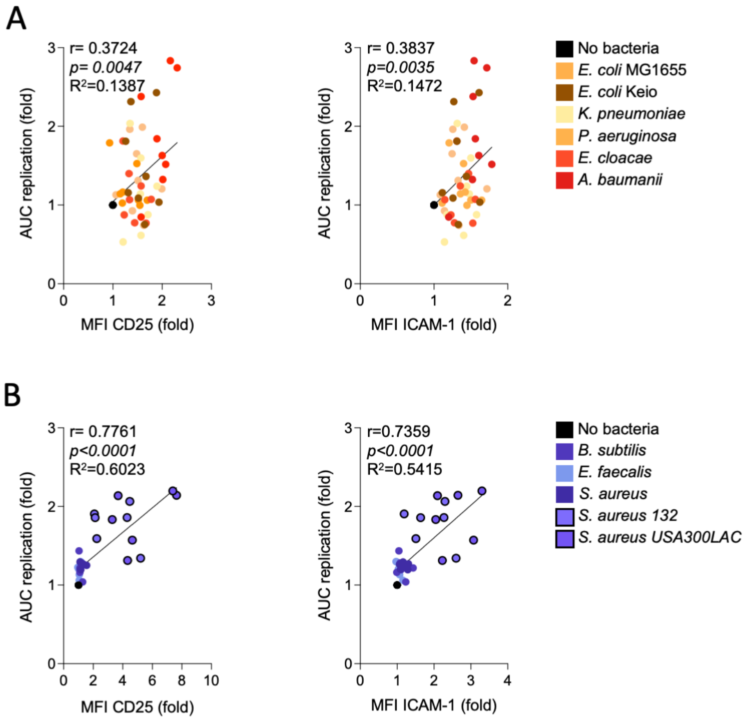

S3 Fig. The extent of CD25 and ICAM-1 upregulation in the presence of bacteria correlates with the extent of viral replication. We calculated the fold induction for CD25, ICAM-1, and viral replication in the presence of bacteria versus the untreated condition, which was set to 1. The data were then plotted to determine the relationship between CD25 or ICAM-1 expression and the observed viral replication. (A) Data for cells that have not been treated or exposed to Gram- bacteria; (B) Data for cells that have not been treated or exposed to Gram+ bacteria. The statistical values of our analysis were determined using correlation and simple linear regression analysis. Pearson "r" coefficient, p value and R squared (R^2) value are indicated in each plot.

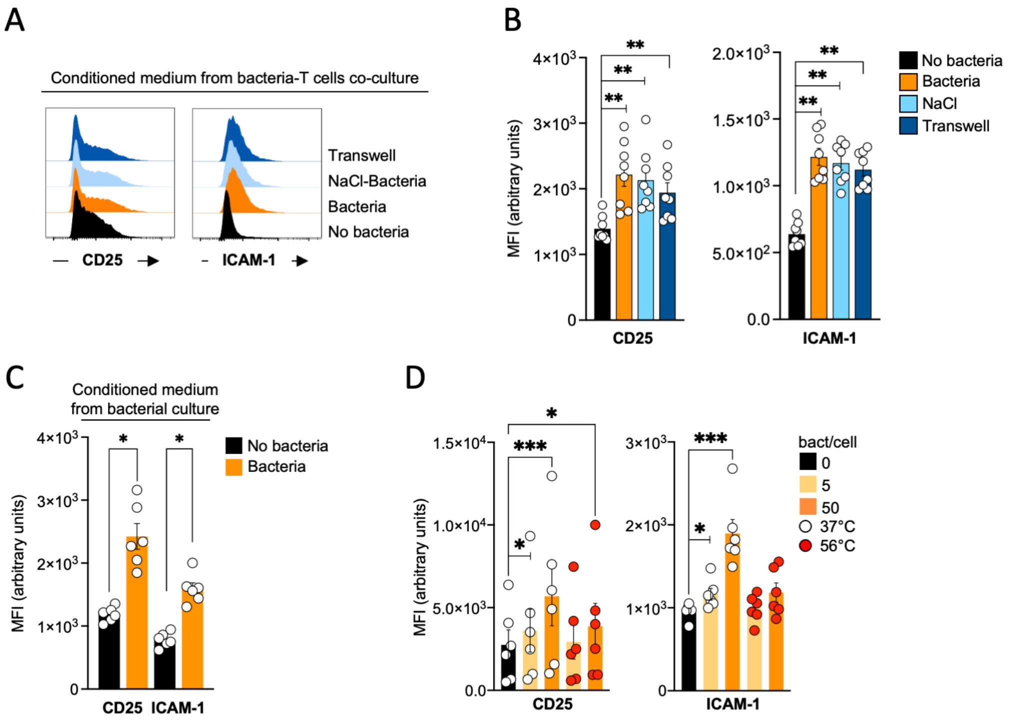

S4 Fig. Bacterial factors that stimulate primary CD4+ T cells are secreted in the supernatant, are not induced by antibiotics, and are produced by metabolically active bacteria. The culture media from Fig 6 B-C were collected, filtered, and used to stimulate new uninfected primary CD4+ T cells. Flow cytometry was used to assess the expression of CD25 and ICAM-1 forty-height hours later. (A) Representative dot plots of the analysis, and (B) Mean +/- SEM of the MFI for each donor tested. The number within the dot plots represents the proportion of positive cells. Each dot in the histograms represents a separate donor. (C) Bacteria were grown overnight in the absence of antibiotics in the culture medium of T cells. The medium was filtered the next day and was used to stimulate uninfected activated primary CD4 T cells after being diluted 1:10 in fresh medium. Flow cytometry was used to evaluate the expression of CD25 and ICAM-1 two days later. Histograms depict the mean +/- SEM of the MFI calculated for each donor. Each donor was tested for every condition. (D) Primary CD4+ T cells were left untreated or cultivated in presence of bacteria that have been grown at 37 °C (white dots) or heat-inactivated for one hour at 56 °C (red dots). Two days after, CD25 and ICAM-1 expression have been evaluated by flow cytometry. Histograms represent the mean +/- SEM of the MFI of CD25 and ICAM-1. Each symbol represents a separate donor. Every donor has been tested for every condition. The Wilcoxon paired t-test was used to calculate statistical differences. *=p≤0.05; **=p≤0.01; ***=p≤0.001.

S4 Fig.

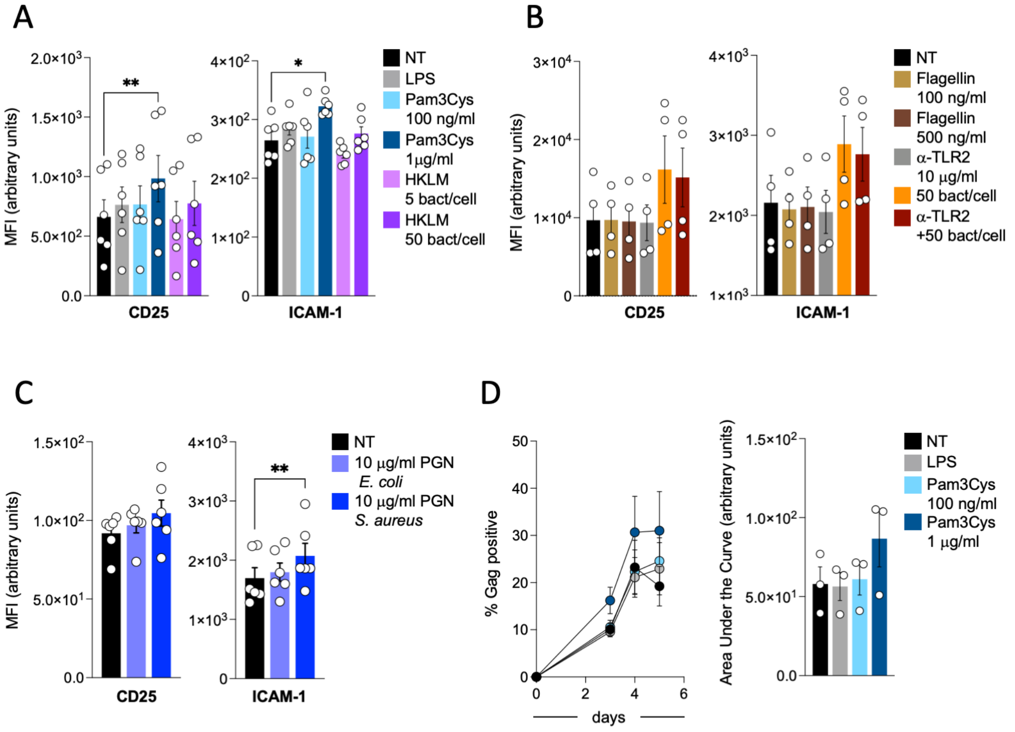

S5 Fig. PRR's agonists do not promote CD25 and ICAM-1 upregulation nor stimulate viral replication. Uninfected primary CD4 T cells were left untreated of incubated in presence of (A) LPS (1 µg/ml), Pam3Cys (100 ng/ml or 1 µg/ml), heat-killed *L. monocytogenes* (HKLM; 5 or 50 bacteria/cell); (B) Flagellin (100 or 500 ng/ml), anti-human TLR2 (10 µg/ml), *E. coli* (50 bacteria/cell), *E. coli* + anti-human TLR2; (C) *E. coli* or *S. aureus* peptidoglycans (PGN, 10 µg/ml). Flow cytometry was used to assess the expression of CD25 and ICAM-1 forty-eight hours later. Histograms in (A-C) represents the mean +/- SEM of the MFI. (D) Primary CD4 T cells were infected with the NL4-3 virus and left untreated or treated with the indicated compounds. Viral replication was analyzed over a week by following the appearance of Gag+ cells in culture by flow cytometry and the area under the curve calculated to compare the different conditions. Each dot represents the value for a donor in an experiment. The Friedman paired t-test was used to calculate statistical differences. *=p≤0.05; **=p≤0.01.

S5 Fig.

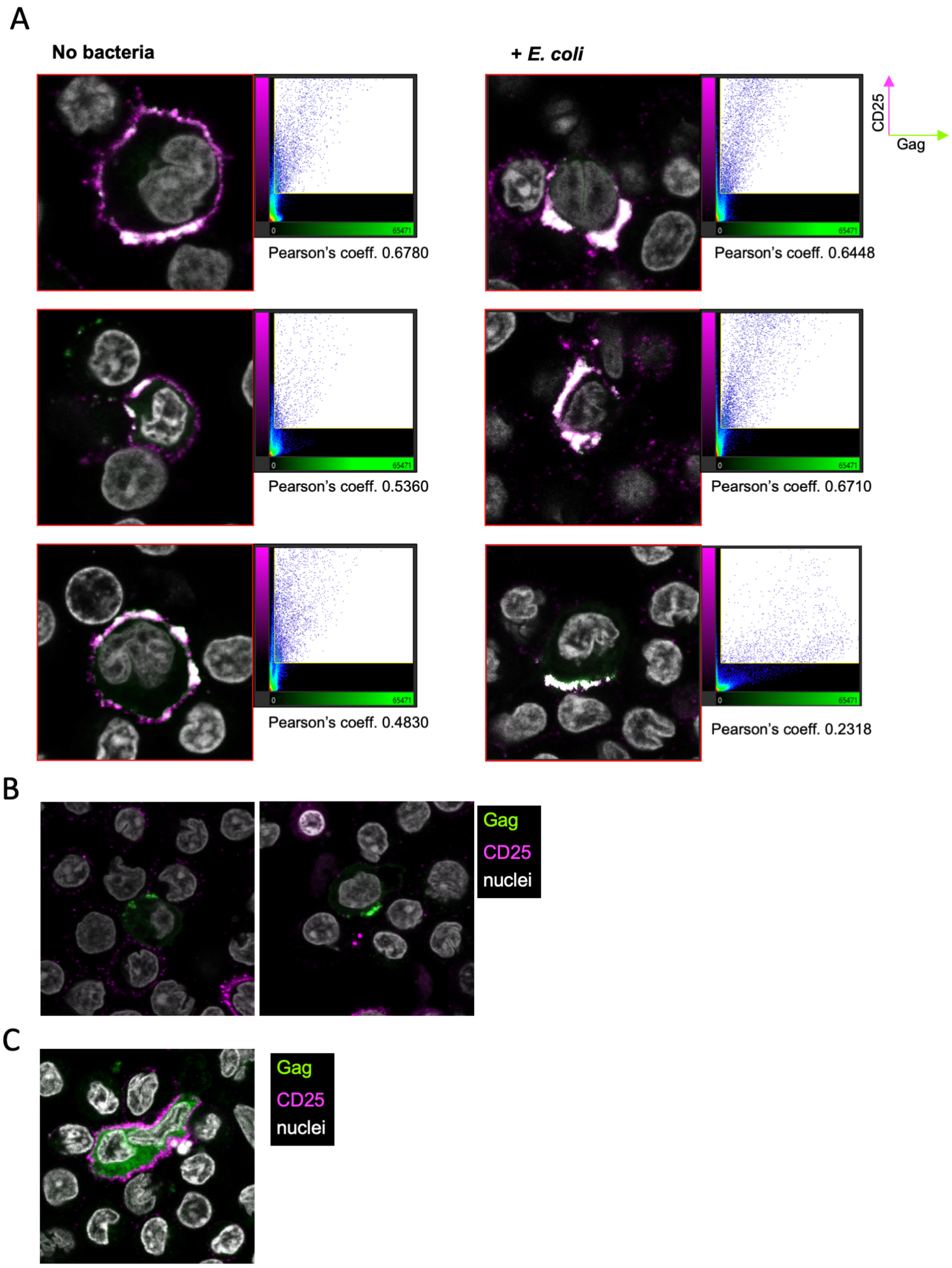
A
No bacteria
+ E. coli
CD25
Gag
Pearson's coeff. 0.6780
Pearson's coeff. 0.6448
Pearson's coeff. 0.5360
Pearson's coeff. 0.6710
Pearson's coeff. 0.4830
Pearson's coeff. 0.2318
B
Gag
CD25
nuclei
C
Gag
CD25
nuclei

S6 Fig.

S6 Fig. CD25 polarizes with HIV-1 Gag at the virological synapse. Primary CD4+ T cells infected with the NL4-3 virus and used as donor cells to form virological synapses with uninfected target cells. Donor and targets were left untreated or exposed to *E. coli* MG1655 for forty-eight hours before to establish the co-culture. CD25 was stained at the cell-surface and the cells were permeabilized to visualize HIV-1 Gag. (A) Virological synapses. The co-localization between CD25 and Gag is shown, together with the dot plot relative to the fluorescence intensity for CD25 and Gag. Pearson's coefficient is also indicated for each image. (B) Two examples of virological synapses where Gag is polarized in absence of CD25 expression.

SUPPLEMENTARY REFERENCES

1. Mutlu EA, Keshavarzian A, Losurdo J, Swanson G, Siewe B, Forsyth C, French A, Demarais P, Sun Y, Koenig L, Cox S, Engen P, Chakradeo P, Abbasi R, Gorenz A, Burns C, Landay A. 2014. A compositional look at the human gastrointestinal microbiome and immune activation parameters in HIV infected subjects. PLoS Pathog 10:e1003829.
2. Garcia C, Chincha O, Leon M, Iglesias D, Barletta F, Mercado E, Ochoa T. 2010. High frequency of diarrheagenic Escherichia coli in human immunodeficiency virus (HIV) patients with and without diarrhea in Lima, Peru. Am J Trop Med Hyg 82:1118-20.
3. Gassama-Sow A, Sow PS, Gueye M, Gueye-N'diaye A, Perret JL, M'Boup S, Aidara-Kane A. 2004. Characterization of pathogenic Escherichia coli in human immunodeficiency virus-related diarrhea in Senegal. J Infect Dis 189:75-8.
4. Guyer MS, Reed RR, Steitz JA, Low KB. 1981. Identification of a sex-factor-affinity site in E. coli as gamma delta. Cold Spring Harb Symp Quant Biol 45 Pt 1:135-40.
5. Baba T, Ara T, Hasegawa M, Takai Y, Okumura Y, Baba M, Datsenko KA, Tomita M, Wanner BL, Mori H. 2006. Construction of Escherichia coli K-12 in-frame, single-gene knockout mutants: the Keio collection. Mol Syst Biol 2:2006 0008.
6. Yang J, Tang Q, Qi T, Chen J, Ji Y, Tang Y, Wang Z, Song W, Xun J, Liu L, Shen Y, Zhang R, Lu H. 2018. Characteristics and Outcomes of Acinetobacter baumannii Infections in Patients with HIV: A Matched Case-Control Study. Sci Rep 8:15617.
7. Tumbarello M, Tacconelli E, Caponera S, Cauda R, Ortona L. 1995. The impact of bacteraemia on HIV infection. Nine years experience in a large Italian university hospital. J Infect 31:123-31.
8. Manfredi R, Nanetti A, Valentini R, Chiodo F. 2001. Acinetobacter infections in patients with human immunodeficiency virus infection: microbiological and clinical epidemiology. Chemotherapy 47:19-28.
9. Nguyen Thi PL, Yassibanda S, Aidara A, Le Bouguenec C, Germani Y. 2003. Enteropathogenic Klebsiella pneumoniae HIV-infected adults, Africa. Emerg Infect Dis 9:135-7.
10. Salami AK, Olatunji PO, Oluboyo PO, Akanbi AA, 2nd, Fawibe EA. 2006. Bacterial pneumonia in the AIDS patients. West Afr J Med 25:1-5.
11. Favre-Bonte S, Joly B, Forestier C. 1999. Consequences of reduction of Klebsiella pneumoniae capsule expression on interactions of this bacterium with epithelial cells. Infect Immun 67:554-61.
12. Gori A, Tincati C, Rizzardini G, Torti C, Quirino T, Haarman M, Ben Amor K, van Schaik J, Vriesema A, Knol J, Marchetti G, Welling G, Clerici M. 2008. Early impairment of gut function and gut flora supporting a role for alteration of gastrointestinal mucosa in human immunodeficiency virus pathogenesis. J Clin Microbiol 46:757-8.
13. Holloway BW. 1955. Genetic recombination in Pseudomonas aeruginosa. J Gen Microbiol 13:572-81.
14. Sharma A, Mong MS, Minamoto G. 2002. Enterococcus faecalis: an unusual cause of meningitis in HIV-infected patients. AIDS Read 12:540-2.
15. Williamson R, Gutmann L, Horaud T, Delbos F, Acar JF. 1986. Use of penicillin-binding proteins for the identification of enterococci. J Gen Microbiol 132:1929-37.
16. Spizizen J. 1958. Transformation of Biochemically Deficient Strains of Bacillus Subtilis by Deoxyribonucleate. Proc Natl Acad Sci U S A 44:1072-8.

17. Senthilkumar A, Kumar S, Sheagren JN. 2001. Increased incidence of Staphylococcus aureus bacteremia in hospitalized patients with acquired immunodeficiency syndrome. Clin Infect Dis 33:1412-6.
18. Nelson LS, Davis SR, Humble RM, Kulhavy J, Aman DR, Krasowski MD. 2015. Impact of add-on laboratory testing at an academic medical center: a five year retrospective study. BMC Clin Pathol 15:11.
19. Valle J, Toledo-Arana A, Berasain C, Ghigo JM, Amorena B, Penades JR, Lasa I. 2003. SarA and not sigmaB is essential for biofilm development by Staphylococcus aureus. Mol Microbiol 48:1075-87.
20. Vergara-Irigaray M, Valle J, Merino N, Latasa C, Garcia B, Ruiz de Los Mozos I, Solano C, Toledo-Arana A, Penades JR, Lasa I. 2009. Relevant role of fibronectin-binding proteins in Staphylococcus aureus biofilm-associated foreign-body infections. Infect Immun 77:3978-91.
21. Kennedy AD, Otto M, Braughton KR, Whitney AR, Chen L, Mathema B, Mediavilla JR, Byrne KA, Parkins LD, Tenover FC, Kreiswirth BN, Musser JM, DeLeo FR. 2008. Epidemic community-associated methicillin-resistant Staphylococcus aureus: recent clonal expansion and diversification. Proc Natl Acad Sci U S A 105:1327-32.
